# Upcycling CO_2_ and PET Waste: Ampere-Level Formate Electrosynthesis in an Integrated Electrolyzer

**DOI:** 10.1021/jacs.5c11708

**Published:** 2025-10-30

**Authors:** Xin Yu, Hesamoddin Rabiee, Abhijit Dutta, Yaqiang Li, Zsolt Szakály, Soma Vesztergom, Lucas Warmuth, Alain Rieder, Peter Broekmann

**Affiliations:** † Department of Chemistry, Biochemistry and Pharmaceutical Science, 27210 University of Bern, Freiestrasse 3, 3012 Bern, Switzerland; ‡ NCCR Catalysis, 27210 University of Bern, Freiestrasse 3, Bern, 3012 Switzerland; § Institute of Molecular Engineering Plus, College of Chemistry, 12423 Fuzhou University, Fuzhou 350108, China; ∥ MTA−ELTE Momentum Interfacial Electrochemistry Research Group, 54616 Eötvös Loránd University, Pázmány Péter sétány 1/A, H-1117 Budapest, Hungary; ⊥ Institute of Catalysis Research and Technology, 150232 Karlsruhe Institute of Technology (KIT), Hermann-von-Helmholtz-Platz 1, D-76356 Karlsruhe, Germany

## Abstract

The rising accumulation of poly­(ethylene terephthalate) (PET) waste and atmospheric CO_2_ presents serious environmental and health challenges. Herein, we introduce a novel strategy for the simultaneous electrochemical upcycling of PET and CO_2_ in a single integrated electrolyzer, enabling ampere-level coproduction of formate. Leveraging careful electrode design of three-dimensional Ni foam at the anode for ethylene glycol (EG, derived from PET hydrolysis) electrolysis, formate formation at 1.2 A cm^–2^ was achieved—outperforming all reported performances for non-noble metal catalysts. A Bi_2_O_2_CO_3_-based gas diffusion electrode (GDE) enabled the selective reduction of CO_2_ (CO_2_RR) to formate at the cathode. By prioritizing enhanced reactant transport and electrode architecture beyond catalyst discovery, this integrated system achieved 100 h of stable operation at 0.50 A cm^–2^ with Faradaic efficiencies of 93.7% (anode) and 86.0% (cathode). Superior energy efficiency was achieved in the proposed membrane-free electrolyzer, with a cell voltage of 2.91 V at 1.0 A cm^–2^, reducing the input energy by 65% to ca. 0.1 kWh mol^–1^. This study highlights the critical role of anodic reaction choices and electrode engineering strategies in developing integrated electrolyzers with superior performance metrics.

## Graphical abstract

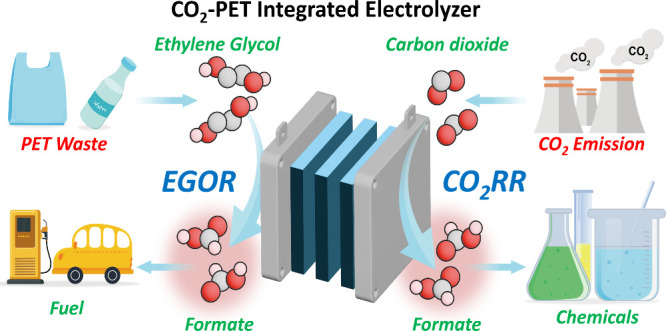

## Introduction

Excessive greenhouse gas emissions and accelerating global warming present an intensifying threat to human society.^1,2^ Among emerging mitigation strategies, electrocatalytic CO_2_ reduction (CO_2_RR) is widely regarded as a highly promising technology.^3^ Driven by renewable electricity, the CO_2_RR enables the production of value-added chemicals such as CO, formic acid, and long-chain alcohols.^4^ Among these, CO and formic acid—two-electron CO_2_ reduction products—have shown high selectivity and production rates at the laboratory scale.^5–7^ Recent techno-economic analyses have reinforced the scalability and economic potential of these products.^8,9^ However, successful implementation hinges on the rational design and optimization of the anodic reaction, which is essential for enhancing the overall efficiency and economic viability of the electrolysis process.

The oxygen evolution reaction (OER) is commonly used as the anodic counterpart of CO_2_RR. Because the OER requires a high onset potential—around 1.23 V vs SHE—and suffers from sluggish kinetics, it consumes more than 90% of the electrical energy input without generating any value-added products.^10^ Therefore, the substitution of the OER with more thermodynamically and kinetically favored reactions has become a research priority. The oxidation reactions of urea, methanol, and ethanol have been extensively investigated,^11–13^ primarily as pairing reactions in high-performance hydrogen production and fuel cells, but they are still not the preferred options for maximizing the value addition of the overall process. In contrast, small molecules derived from low-cost biomass^14^ or plastic waste^15^ offer a dual benefit: enabling waste valorisation, while generating valuable products, thereby enhancing the overall efficiency of process.

Ethylene glycol (EG), a hydrolysis product of poly­(ethylene terephthalate) (PET), has recently gained attention as a potential anodic feed. PET, widely used in packaging and textiles with an annual global consumption of 28.45 Mt, has a recycling rate of only 23%, highlighting the need for more sustainable end-of-life solutions.^16^ Mechanical PET recycling is energy-intensive and degrades the quality of the recycled products. Whereas, hydrolysis depolymerizes PET back to virgin monomers for recycling and even upcycling.^17^ Duan et al. recently proposed a hydrolysis-electrolysis coupling strategy to address this challenge.^18^ PET was hydrolyzed under alkaline conditions into its monomers, terephthalic acid (TPA) and EG. Subsequently, EG was electrochemically oxidized into formate to address challenges of downstream separation. This integrated process delivered profits of up to $355 per ton of PET, with formate accounting for two-thirds of the total revenue-exceeding the contribution of H_2_ production at the cathode.^18^

The studies on the CO_2_RR+EGOR integration often suffer from low current density and insufficient stability. In the case of ethylene glycol oxidation reaction (EGOR), nickel-based catalysts have shown promising selectivity toward formate production by effectively cleaving C–C bonds while avoiding complete mineralization.^19,20^ Various strategies, such as doping and nanoengineering, have been reported for catalyst design in this scenario. For instance, introducing cobalt into the Co–Ni_3_N catalyst facilitated the redox behavior of Ni^2+^/Ni^3+^ couple, thereby achieving an ultralow EGOR onset potential (1.15 V vs RHE).^21^ However, (pre)­catalyst reconstruction under bias was inevitable, which led to etching of doping components (N, S, B, etc.) and the gradual degradation of fine-tuned morphology over time.^21,22^

Equally critical to catalyst development is electrode structural engineering, especially under high current densities, where mass transport limitations become increasingly significant. Since the diffusion layer typically exists on a micrometer scale, the rapid consumption of surface reactants can significantly hinder the effective exposure and utilization of active sites. It becomes particularly severe for catalysts with high surface areas, amplifying the discrepancy between their intrinsic activity and practical current output. 3D metal-foam-based electrodes are advantageous to provide abundant surface area, but careful design of their porous structure is required to mitigate the diffusion limitation. The open macroporous morphology facilitates efficient reactant transport into the interior of the 3D catalyst, thereby enabling optimal use of the high surface density of reactive sites. Ampere-level current and robust stability have been demonstrated in both CO_2_RR and nitrate electroreduction (NO_3_RR) systems.^23–25^

In the CO_2_–PET dual electrolysis concept, formate is the preferred product at both electrodes, enabling coproduction with strong industrial potential due to its scalability and the simplicity of product separation.^26,27^ Our earlier work demonstrated the potential of Bi_2_O_2_CO_3_ as a stable and formate-selective catalyst for CO_2_ electroreduction.^28^ Concurrently, gas diffusion electrodes (GDEs) have become the dominant architecture for continuous CO_2_ feed.^29,30^ However, challenges such as electrolyte flooding and carbonation,^31^ demand careful electrode engineering,^32,33^ as failure of the cathodic GDE can undermine the stability and performance of the entire integrated system.

In this study, beyond catalyst development, we develop an integrated CO_2_RR+EGOR system with accelerated reactant transport and engineered electrodes to unlock ampere level formate coproduction. A 3D Ni-based foam with an engineered pore structure and abundant active area was used as the anode for EGOR. Its tunable open macroporous structure allows for a superior catalytic performance, achieving a Faradaic efficiency (FE) of 62.6% for formate at the record-high current density of 1.2 A cm^–2^. For the cathodic CO_2_RR, a highly stable Bi_2_O_2_CO_3_-based GDE (denoted herein after bismuthoxycarbonate: BOC) was fabricated by incorporating hydrophobic PTFE particles into the catalyst layer (CL). Careful electrode engineering enabled the integrated CO_2_RR+EGOR configuration to deliver significantly enhanced performance—51% higher formate productivity and 46% lower energy demand—than the conventional CO_2_RR+OER setup. Notably, this study presents the first experimental demonstration of a membrane-free electrolyzer for formate coproduction, achieving improved energy efficiency. The *operando* Raman spectroscopy and diffusion modeling were conducted, revealing the role of Ni active species and engineered catalyst porosity in facilitating efficient mass transport. This work provides novel insights into efficient waste valorization through strategic engineering of electrodes and electrolyzers beyond catalyst advances.

## Results and Discussion

### Electrode Engineering for Anode and Cathode

Figure 1a depicts the custom-built flow electrolyzer used in this study for the formate coproduction. To ensure efficient reactant-catalyst contact for liquid-phase oxidation at the anode, a flow-through electrode architecture with a highly active/selective catalyst material and an augmented surface area was employed. We developed a highly porous 3D Ni foam with engineered pore structure through dynamic hydrogen bubble templating (DHBT) method,^23^ as illustrated in Figure 1b. At an optimal electrodeposition current density of −3 A cm^–2^, hydrogen evolution and nickel deposition occurred concurrently at the electrode–electrolyte interface. The evolution of hydrogen bubbles disrupted uniform Ni growth, thereby serving as dynamic templates for the formation of a porous Ni foam. The process of bubble nucleation, growth, and detachment at the electrode surface during metal deposition resulted in a hierarchical porous structure within the 3D Ni foam. The DHBT process was performed using a three-electrode setup (Scheme S1), as described in our earlier works.^23,24^

**Figure 1:**
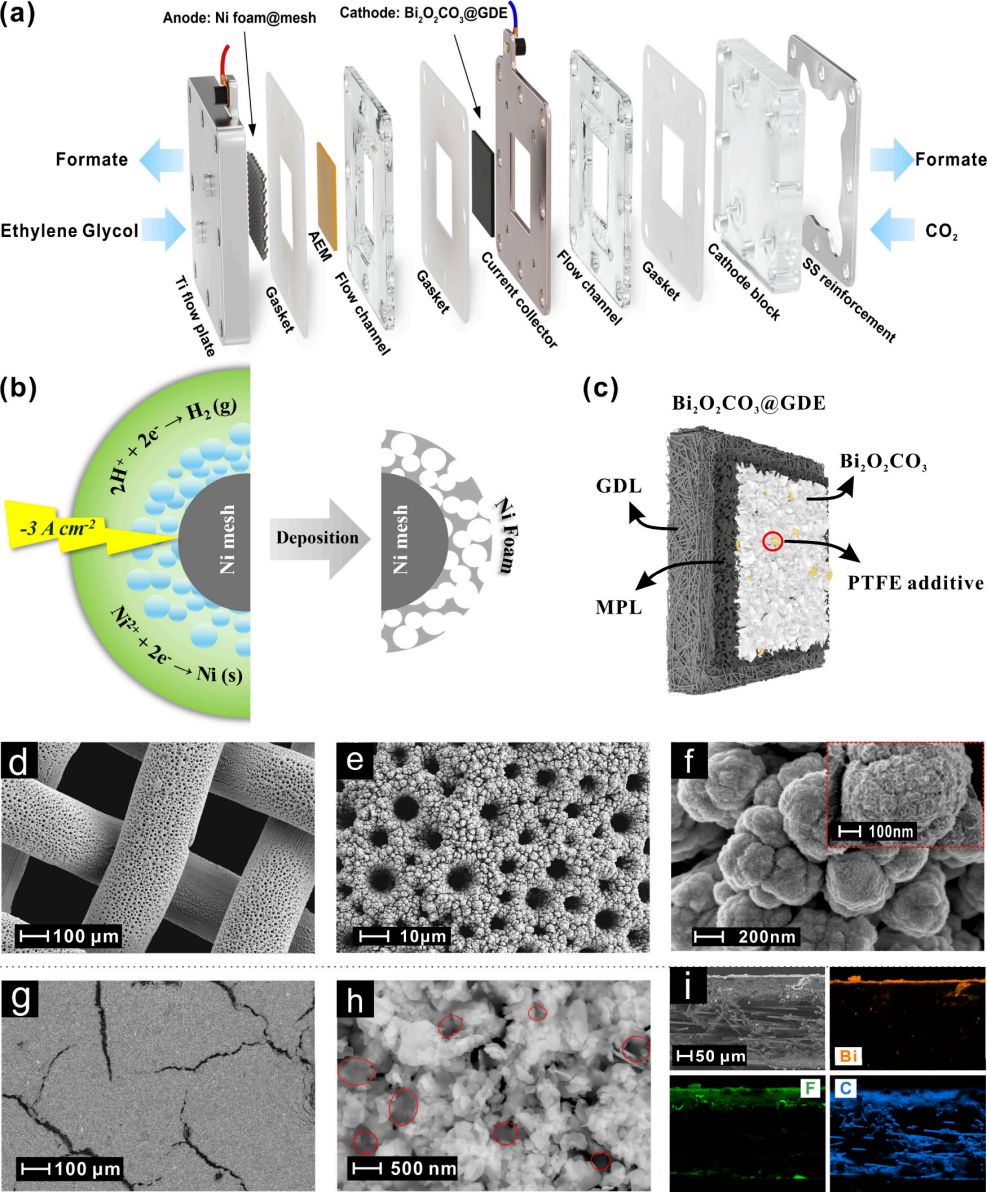
Schematic of (a) the flow electrolyzer in this study, (b) the dynamic hydrogen bubble template (DHBT) method for 3D Ni foam fabrication as anode for EGOR, and (c) the structure of Bi_2_O_2_CO_3_@GDE used for CO_2_RR, where MPL and GDL refer to microporous layer and gas diffusion layer, respectively (MPL: Microporous layer, GDL: Gas-diffusion layer, PTFE: Polytetrafluoroethylene). The surface SEM images of (d-f) 3D Ni foam deposited on Ni mesh and (g,h) Bi_2_O_2_CO_3_@GDE. The gray frosted areas marked with red circles are PTFE microflakes due to their insulating properties. (i) Cross-section SEM image and corresponding EDX mapping of Bi_2_O_2_CO_3_@GDE for the elements of Bi, F and C.

As demonstrated in Figure 1d and e, a uniform porous Ni foam structure was formed on the Ni mesh substrate after 30 s of deposition, with surface pore diameters ranging from approximately 3.8 to 9.4 μm. The pore size was strongly influenced by the hydrogen bubble templates, which tended to agglomerate into larger and fewer bubbles as they grew and migrated away from the electrode surface (Figure S1). By varying the deposition duration from 5 to 60 s, hierarchical porosity ranging from 96.1% to 97.5% was achieved (Figure S2). High-magnification SEM images (Figure 1f) revealed that the Ni foam exhibited a cauliflower-like morphology composed of packed Ni clusters. Notably, petal-like nanolayers were observed on the surface of these clusters, which were identified as NiO via Raman (Figure S3) and XPS (Figure S4) spectroscopy.^34^ Such a petal-like nano structure was not seen for the Ni deposition on a planar foil substrate (Figure S5), likely due to differences in current distribution induced by substrate geometry, as well as local concentration gradients near the substrate surface. Despite morphological differences, both foil and mesh supported Ni foams exhibited similar catalyst compositions (Figure S3) and electrochemical characteristics (Figure S6). The flow-through configuration of 3D Ni foam grown on mesh not only enhances solution turbulence to replenish reactant availability but also increases catalyst–electrolyte contact, thereby improving catalyst utilization.

Cross-sectional SEM analysis (Figure S7) indicated that the Ni foam thickness increased from 4.1 μm at 5 s to 84 μm at 60 s of deposition time. While the resulting thick and porous catalyst layer provides a large surface area, it also introduces mass transport limitations, which can complicate the determination of the electrochemical active surface area (ECSA), particularly when using Faradaic methods. To address this, dimethyl viologen dichloride (DMVCl_2_) was employed as a redox probe for ECSA evaluation.^24^ The ECSA of the 3D Ni foams, normalized to blank Ni foil, ranged from 2.2 to 4.8 cm^2^/cm^2^, substantially lower than values obtained using the classical double-layer capacitance method (Figures S8, S9, and S10a). Similar measurements on the mesh-based Ni foam with a thickness of 21.2 μm yielded an ECSA of 2.4 cm^2^/cm^2^. Moreover, the mass contribution to ECSA exhibited opposite trends between Faradaic (DMVCl_2_ redox) and non-Faradaic (capacitance) methods (Figure S10b). The increasing contribution to the “capacitive ECSA” implied the geometric surface area of Ni foam benefiting from higher porosity in longer deposition durations, while it failed to convert into a “Faradaic ECSA”. This discrepancy can originate from mass transfer limitations, where species involved in the Faradaic process (other than those water-derived) are massively consumed before reaching the catalyst at depth, so inner parts of the catalyst cannot make an effective contribution to Faradaic current. Essentially, only catalyst regions directly accessible to reactants contribute to Faradaic processes—particularly critical in 3D porous architectures—yet this distinction between capacitive and Faradaic ECSA remains largely underexplored in the current literature. This hypothesis is further supported by the electrolysis results discussed later.

To ensure electrolyzer stability, cathodic electrode design must also be carefully optimized. Gas diffusion electrodes (GDEs) for CO_2_RR often suffer from stability challenges due to flooding and/or carbonation. In this study, GDEs were fabricated by airbrushing a catalyst ink onto the microporous layer (MPL) (Figure 1c and Scheme S2), with PTFE microflakes incorporated into the ink to enhance the hydrophobicity and mitigate flooding. Bi_2_O_2_CO_3_ (BOC) nanosheets were chosen as the CO_2_RR catalyst based on its previously demonstrated superior stability and activity for formate production.^28^ As shown in Figure 1g, the BOC catalyst was uniformly distributed across the GDE surface, although visible cracks originating from the underlying MPL were observed. Figure 1h shows the successful integration of PTFE particles (size of 1 μm) into the catalyst layer (CL), contributing to increased hydrophobicity and improved resistance to flooding (Figures S11 and S12). XRD analysis (Figure S13b) verified that the BOC retained its crystalline structure (PDF #84–1752) after deposition onto the GDE. Cross-sectional imaging (Figure 1i) revealed a 3–5 μm thick BOC layer covering the MPL. Fluorine detected by EDS was mainly attributed to PTFE within the CL and Nafion ionomer infiltration into the MPL.

### Electrocatalytic Performance of Integrated Electrolyzer

The electrolysis was conducted galvanostatically from 0.2 to 1.2 A, 30 min for each current point in the electrolyzer (Figure 1a). Note that geometric surface areas of anode and cathode were 1 cm^2^ and 6.25 cm^2^, respectively, to enable the broad current window of anode and ensure adequate stability of the cathode. Benefiting from the active 3D Ni foam, the FE to formate (FA) started at 95% at 0.2 A and remained as high as 62.6% at 1.2 A (Figure 2a). Glycolate (GA) as the main byproduct accounted for 7–13% FE of the EG oxidation. Simultaneously, on the cathode side, formate was produced from CO_2_ reduction, with an FE over 92%. Considering the formate crossover through anion exchange membrane (AEM), the total FE of the cathode was compensated to 100%, based on the raw data (Figure S14). The presumed migration from cathodic formate yield was subtracted from the total yield to accurately determine the FE of the anodic counterpart. It is further confirmed by the results from CO_2_RR+OER experiments in Figure 2b. At 1.2 A, formate FEs of 62.6% and 92.4% from the EG oxidation and CO_2_ reduction, respectively, were simultaneously achieved in the paired electrolyzer, indicating the capability of the engineered anode to reach ultrahigh current densities while producing formate as the major product. As compared with OER, as the typical anodic reaction coupled with CO_2_RR, a greater formate productivity was achieved by integrating CO_2_RR with EGOR. As shown in Figure 2c, the formate formation increased by over 50% (30.1 vs 19.6 mmol h^–1^ at 1.2 A). The presence of ethylene glycol reduces the anodic potential, thereby decreasing the overall cell voltage in the CO_2_RR+EGOR system. At 1.0 A, the cell voltage dropped from 8.02 V (with OER) to 6.52 V (with EGOR) (Figure S15), resulting in 46.3% reduction in energy consumption (Figure 2d). Overall, precise engineering of both anode and cathode electrodes enabled ampere-level formate coproduction as the major product with significantly improved energy efficiency.

**Figure 2:**
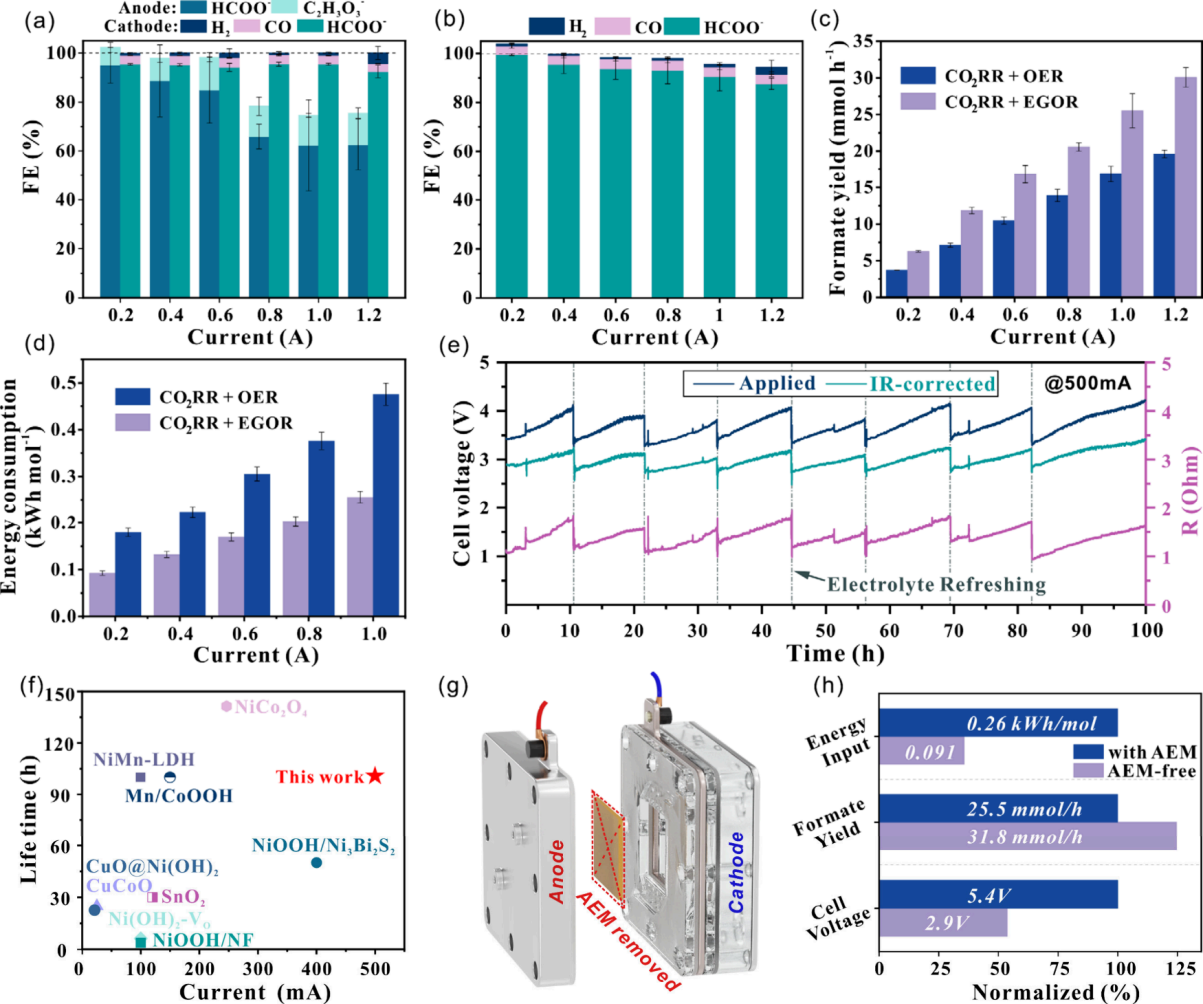
Faradaic efficiency for CO_2_RR integrated with (a) EGOR and (b) OER in the flow cell, (c) formate yield and (d) energy consumption in CO_2_RR+OER and CO_2_RR+EGOR configurations, respectively. (e) Cell voltage and resistance profiles of the CO_2_RR+EGOR system during the stability test at 500 mA, with average formate FEs of 93.7% (anode) and 86.0% (cathode). (f) Stability comparison of integrated CO_2_RR+EGOR electrolysis (see Table S2). (g) Schematic of the AEM-free electrolyzer and (h) corresponding electrolysis key results. Note that the geometric surface areas of Ni foam and BOC@GDE are 1.0 cm^2^ and 6.25 cm^2^, respectively, for all the measurements in the flow cell.

The stability of the integrated configuration was evaluated via long-term electrolysis at 500 mA cm^–2^ for 100 h. During this process, the cell voltage gradually increased from 3.4 to 4.0 V, but consistently returned to the initial value upon electrolyte replenishment (Figure 2e). Moreover, online high-frequency impedance (100 kHz, sine amplitude of 1 mA) was applied to probe the real-time resistance during the stability test. The resistance—primarily originating from the electrolytes—fluctuated synchronously with cell voltage, indicating substantial OH^–^ consumption under high-current conditions (according to R1–5 in SI). With the operando impedance, the cell voltage can be decoupled and analyzed by *iR*-correction (the *iR*-correction was only calculated but not compensated for during the galvanostatic electrolysis). The *iR*-corrected voltage exhibited a reduced and stabilized profile (2.8–3.2 V), where the residual fluctuation was primarily attributed to EG depletion. Importantly, throughout the 100 h electrolysis at 500 mA cm^–2^, the FEs of formate remained high and stable, averaging 93.7% at the anode and 86.0% at the cathode (Figure S16). These results demonstrated both the Ni foam and BOC@GDE maintain their high activity and stability during the paired electrolysis.

Postelectrolysis SEM imaging revealed that the Ni foam retained its macroporous structure after 100 h of operation, confirming the morphological stability under anodic conditions at high current densities (Figure S17a-c). XPS analysis confirmed that the surface of Ni foam was dominated by divalent nickel species, with O 1s spectra further verifying the coexistence of NiO and Ni­(OH)_2_.^35^ This surface composition remained stable after electrolysis (Figure S4). Moreover, as characterized by TEM (Figure S18), the amorphous NiO layers grew laterally along the 2D plane after electrolysis. It can also validate the high-frequency turnover of Ni^2+^/Ni^3+^ redox in the EGOR process.^36^ At a higher current density of 1.0 A cm^–2^, the Ni foam exhibited stable performance for 6 h, after which the cell voltage increased sharply from 5.34 to 10 V (Figure S19), indicating catalyst deactivation. SEM analysis confirmed the loss of foam morphology due to evolution of nickel oxide (Figure S17d-f), underscoring the need to define optimal operating conditions.

For the cathode GDE, long-term CO_2_RR revealed formation of the microspheres composed of nanosheets, as seen in postelectrolysis SEM images (Figure S20). HRTEM images of the postelectrolysis catalyst (Figure S21) showed the same lattice spacing as the pristine sample corresponding to the (121) plane of Bi_2_O_2_CO_3_ (PDF #84–1752), but with reduced crystallinity. The chemical composition of the Bi_2_O_2_CO_3_ catalyst remained unchanged, as confirmed by XRD and Raman analyses (Figure S13). However, localized K_2_CO_3_ precipitations were observed at near-surface cracks in the GDE—identified by EDX (Figure S20)—consistent with our previously reported findings.^37,38^ With Bi_2_O_2_CO_3_/GDE maintaining its structural integrity, CO_2_ reduction in the integrated electrolyzer showed stable performance throughout the long-term test. The FEs remained consistently at 86.0% for formate, 8.1% for CO, and 5.8% for H_2_, evidencing the stability of the GDE during 100 h of continuous operation (Figure S16b).

Therefore, through engineering the electrode structure in both the anode and cathode sides, the CO_2_RR and EGOR integrated system exhibited an excellent performance at the high current densities of 0.5 to 1.0 A cm^–2^. The fabricated 3D Ni foam anode demonstrated exceptional performance, outperforming previously reported non-noble metal catalysts for EGOR (Table S1). Moreover, this work achieved ampere-level current density with formate as the major product and remarkable stability, setting a new benchmark in formate coproduction (Table S2 and Figure 2f).

### Membrane-Free Electrolyzer Design

To further reduce the cell voltage and simplify the electrolyzer toward a more industrially viable design, we explored the membrane-free PET-CO_2_ electrolysis for the first time. Moreover, since formate is generated on both the anode and cathode, the use of anion exchange membrane (AEM) becomes less essential in this integrated system. Accordingly, the AEM was removed from the electrolyzer, and 1 M EG + 1 M KOH was used as both anolyte and catholyte (Figure 2g). This resulted in an initial reduction of cell voltage by over 20% (from 5.40 to 4.10 V), due to the decreased cell resistance by removing the membrane (Figure S19b vs Figure S22a). Subsequently, the applied cell voltage gradually increased to 10 V, while the *iR* corrected voltage remained relatively stable after 1 h electrolysis. Online monitoring of cell resistance and pH (Figure S22b) revealed a gradual increase in the resistance accompanied by a pH drop from 13.63 to 13.15 during electrolysis. These changes are consistent with the overall reaction mechanism (R5), where OH^–^ is progressively replaced by HCOO^–^ as the reaction proceeds. Notably, the pH stabilized immediately upon stopping the electrolysis—despite continued reactant feeding—indicating that CO_2_ crossover and other nonelectrochemical effects are not responsible for the observed shifts. Based on this understanding, increasing the KOH concentration to 3.0 M extended the operational lifetime to approximately 2.5 h and lowered the initial cell voltage to 2.9 V (Figures 2h and S22c,d). After 2.5 h of electrolysis, both the anode and cathode exhibited deactivation, leading to a decline in formate productivity (Figure S23). This demonstration highlights the promise of membrane-free design to enable high-current, energy-efficient formate production—extending beyond catalyst and electrode engineering alone. Future work should systematically address electrolyte and pH management, along with the development of stable catalysts, to further enhance the long-term stability of membrane-free configurations.

### Mechanistic Insights into EGOR and PET Hydrolysate Oxidation

We further explored the origin of the exceptional electrocatalytic activity of the as-prepared anode. Cyclic voltammetry (CV) was performed to investigate the electrochemical behavior of Ni foam (Figure 3a). In 1 M KOH, the Ni foam exhibited an onset potential of 1.35 V (versus RHE herein after), corresponding to the oxidation of Ni^2+^ to Ni^3+^ (Ni^0^ to Ni^2+^ transition can occur in the ambient air and KOH electrolyte without applied bias).^39^ With the addition of ethylene glycol, identical onset potential was observed, while the current response increased significantly and exceeded 60 mA cm^–2^ at 1.45 V, demonstrating the excellent oxidation capability of the as-prepared Ni foam. The Ni reduction peak in the backward scan further confirmed that the Ni redox transitions are involved in the EG oxidation. The secondary current increase beyond 1.6 V is attributed to the oxygen evolution reaction (OER). Note that the gap in overpotential (at j = 30 mA cm^–2^) between OER and EGOR is ca. 340 mV, indicating that EGOR is thermodynamically more favorable than OER. Moreover, Tafel analysis can also confirm that EGOR owns the kinetic advantage over OER (Figure 3b). To get the neat kinetic current in the LSV to measure Tafel slopes, we ensured sufficient convective conditions (conducted in a rotating disk electrode (RDE) configuration), slow sweep rate (1.0 mV s^–1^), 100% *iR*-correction, and 100% FE region. With the rotation rates from 1200 to 1600 rpm, only negligible shifts happened to the Tafel slopes, which validates the independence on convectional mass transport (Figures S24 and S25).^40,41^ The Tafel slope dropped significantly from 48 mV dec^–1^ to 10.5 mV dec^–1^ with the addition of 100 mM EG, representing a faster kinetic over the OER (Figure 3b). Moreover, the Tafel slope of EGOR is even lower than that of intrinsic Ni^2+^ oxidation (13 mV dec^–1^), suggesting that EG oxidation could actively facilitate Ni species evolution (Figure S26).

**Figure 3:**
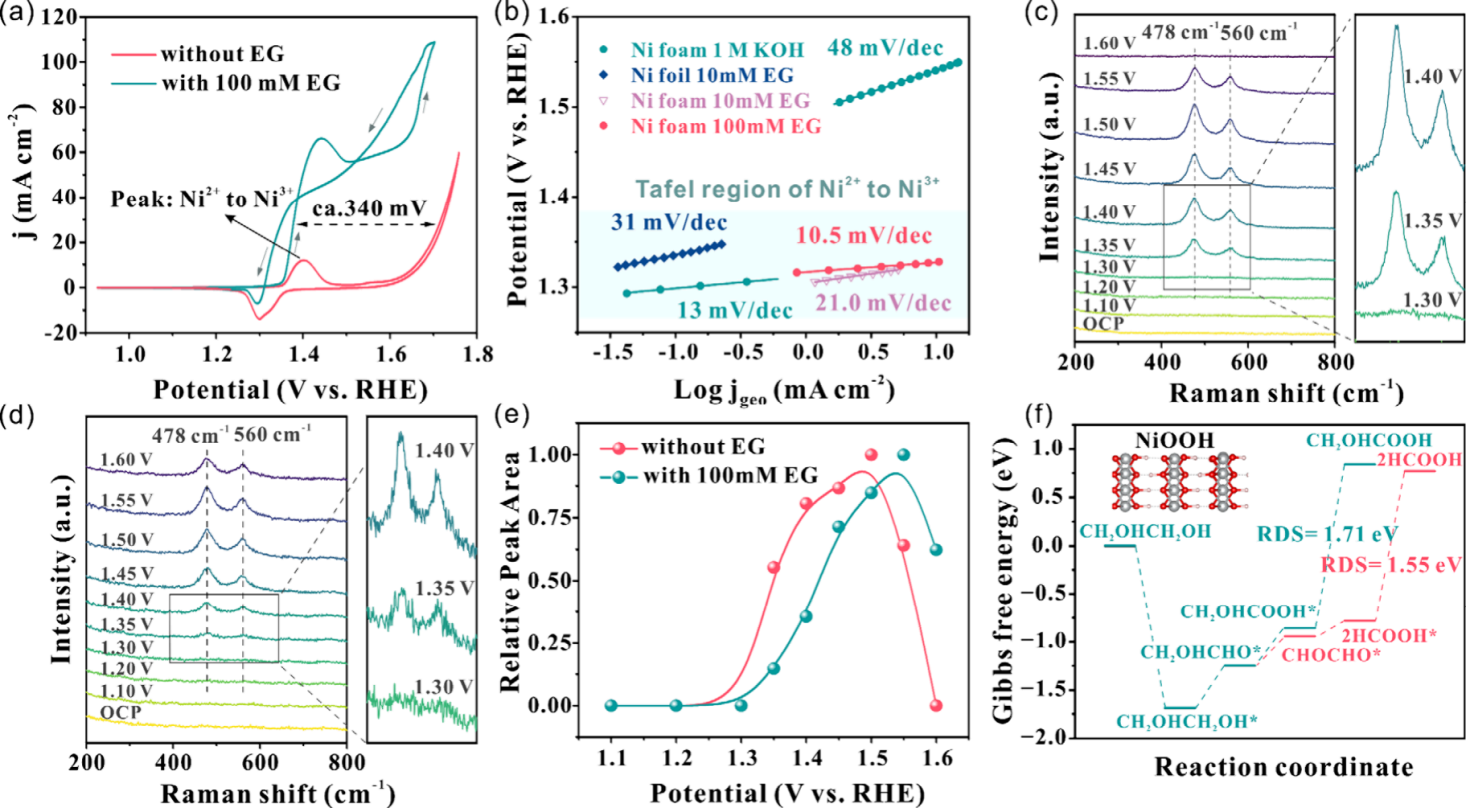
(a) Cyclic voltammetry (CV) of 3D Ni foam with and without EG addition in 1 M KOH, with 10 mV s^–1^ sweep rate and 85% *iR*-correction in H-type cell, (b) Tafel slopes of Ni foil and Ni foam with different EG concentrations, derived from LSV curves from a RDE system, with 1.0 mV s^–1^ sweep rate and 100% *iR*-correction. Potential dependent in situ Raman spectra of (c) without and (d) with 100 mM EG addition in 1 M KOH, and (e) relative peak (at 478 cm^–1^) area. (f) Gibbs free energy diagram of EGOR on NiOOH. The inserted image is crystal structure of NiOOH. See the Supporting Information (Figure S27) for more details of DFT calculations.

Consistent results were observed in the *in situ* Raman analysis (Figure 3c and d), where a potential-dependent approach was employed to monitor the catalyst evolution during ethylene glycol oxidation. When the potential reached 1.35 V, two characteristic peaks emerged at 478 cm^–1^ and 560 cm^–1^, corresponding to the Ni–O bending and stretching vibrations in NiOOH, respectively.^21^ The alignment between the NiOOH excitation potential and the current onset observed in CV strongly supports the role of NiOOH as the active species in EGOR. The Raman spectra in the blank KOH electrolyte exhibited similar peak features, further confirming the presence of NiOOH from 1.35 V onward. Furthermore, it has been reported that ethylene glycol, like other short chain alcohols, undergoes indirect oxidation on NiOOH,^42^ i.e. NiOOH could be reversibly reduced to Ni^2+^ by ethylene glycol (Figure S26). The Raman peaks were integrated, and then the relative peak area was employed as a descriptor for the surface abundance of NiOOH (Figure 3e). In the presence of EG, less abundant NiOOH was detected in potential window of 1.35–1.5 V, being consumed by EG oxidation. This further supports the involvement of NiOOH in the EGOR process via an indirect oxidation mechanism, offering a complementary perspective to the findings reported by Michael et al.^43^ The decline in peak area (after 1.50 V for the case without EG addition, 1.55 V with 100 mM EG) was attributed to oxygen bubbles formation and attachment on the electrode surface, which blocked Raman signals when the potential went into the OER region. Therefore, the addition of ethylene glycol shifted the onset of the OER to more anodic potentials, successfully suppressing the undesired OER process by Ni foam.

DFT calculations were performed to elucidate the reaction pathway and selectivity. The Gibbs free energy profile of EGOR (Figures 3f and S27a) indicates that the reaction proceeds via CH_2_OHCHO* to HCOOH*,^26,44^ as the charge-transfer is more favorable than the alternative pathway from CH_2_OHCHO* to CH_2_OHCOOH* (Figure S27b). The crystal orbital Hamilton population (COHP) analysis further revealed that the bonding energy of HCOOH* (−1.68 eV) is lower than that of CH_2_OHCOOH* (−2.86 eV) on NiOOH,^45^ thereby leading to a high selectivity toward formate (Figure S27c and d). Moreover, the energy barrier for OER (RDS = 2.18 eV) is higher than that for EGOR (RDS = 1.55 eV for formate), highlighting the thermodynamic favorability of EGOR over OER on Ni foam (Figure S27e).^46^

To further confirm the superiority of Ni for EGOR, different transition-metal foams (Co, Cu, and Fe) were fabricated using the DHBT method and compared to Ni foam under identical conditions. The porosities and mass loadings can vary due to their different electrodepositing properties (Figure S28). In this case, ECSA determination using the DMVCl_2_ redox method^24^ (Figure S29) was implemented on every foam and was normalized for subsequent experiments. The overpotential at 10 mA cm^–2^ follows the order of Ni < Cu < Co < Fe (Figure S30), indicating Ni has the most favorable kinetics for EGOR. The polarization curves of Co and Cu foams showed no apparent EG diffusion limitation, suggesting that the current observed at high overpotentials may originate from OER rather than EGOR. The performance was also investigated by potentiostatic electrolysis at 1.5 V vs RHE (Figure S31). All of these foams exhibited high formate selectivity, and the FE_formate_ for Cu foam (84.6%) was slightly higher than that for Ni (80.0%). However, the total and partial current densities of Ni foam were nearly twice those of Cu and substantially exceeded those of Co and Fe (Figure S31b). Overall, the reported 3D Ni foam exhibited the best catalytic performance for EGOR among the investigated transition-metal foams.

Furthermore, to showcase the real-life application of this design, a commercial PET bottle was directly used as the precursor for EGOR. Upon depolymerization and hydrolysis in 3 M KOH, PET is converted into its monomers—ethylene glycol and terephthalate (TPA).^15^ Cyclic voltammetry was conducted to compare the electrocatalytic properties of 3D Ni foam in PET hydrolysate and EG monomer (Figure S32a). The CV curve in the PET hydrolysate exhibited an onset potential (1.37 V) similar to that observed with pure monomers, confirming the catalytic activity of the 3D Ni foam toward direct hydrolysate oxidation. However, the lower peak current reflected incomplete PET depolymerization.^47^ Further studies are required to systematically optimize the hydrolysis parameters, such as temperature, KOH concentration, and reaction time. Moreover, a charge-dependent NMR analysis^44^ (Figures S32b, c) confirmed that the 3D Ni foam effectively oxidized the PET derived EG to formate. This result underscores the feasibility of direct PET hydrolysate oxidation with this system for simultaneous PET and CO_2_ upcycling.

### Insights into Mass Transport Phenomena

The significantly high current density observed for the 3D porous Ni foam arises from improved mass transport and the enhanced utilization of active sites. To isolate transport effects, we compared ethylene glycol oxidation in a static H-cell versus a flow cell under potentiostatic control (constant charge at 360 C to avoid the massive deviation in reactant consumption, 100% *iR*-compensated) from 1.35 to 1.7 V. In the H-cell, FE of formate fell from 98.8% at 1.35 V to 27.7% at 1.7 V, with OER overtaking at above 1.6 V (Figure S33a), consistent with the cyclic voltammetry in Figure 3a, and formate partial currents plateaued between 1.40 and 1.65 V due to the diffusion-limitation condition of H-cell (Figure 4a). In contrast, convective conditions of the flow electrolyzer alleviated these limitations, achieving a maximum of 750 mA cm^–2^ formate partial current density (Figure 2a, at 1.2 A cm^–2^), over twice higher than what was achieved in static conditions (∼300 mA cm^–2^, see Figure S34). This indicates the necessity of tuning the fluid mechanics of solution to fully activate the 3D catalyst. Beyond this, inner foam regions became “reactant-starved” as oxidation outpaced convective replenishment, causing FE loss. These findings demonstrate that sufficient convective transport is essential to unlock all active sites and sustain stable high-current-density performance.

**Figure 4:**
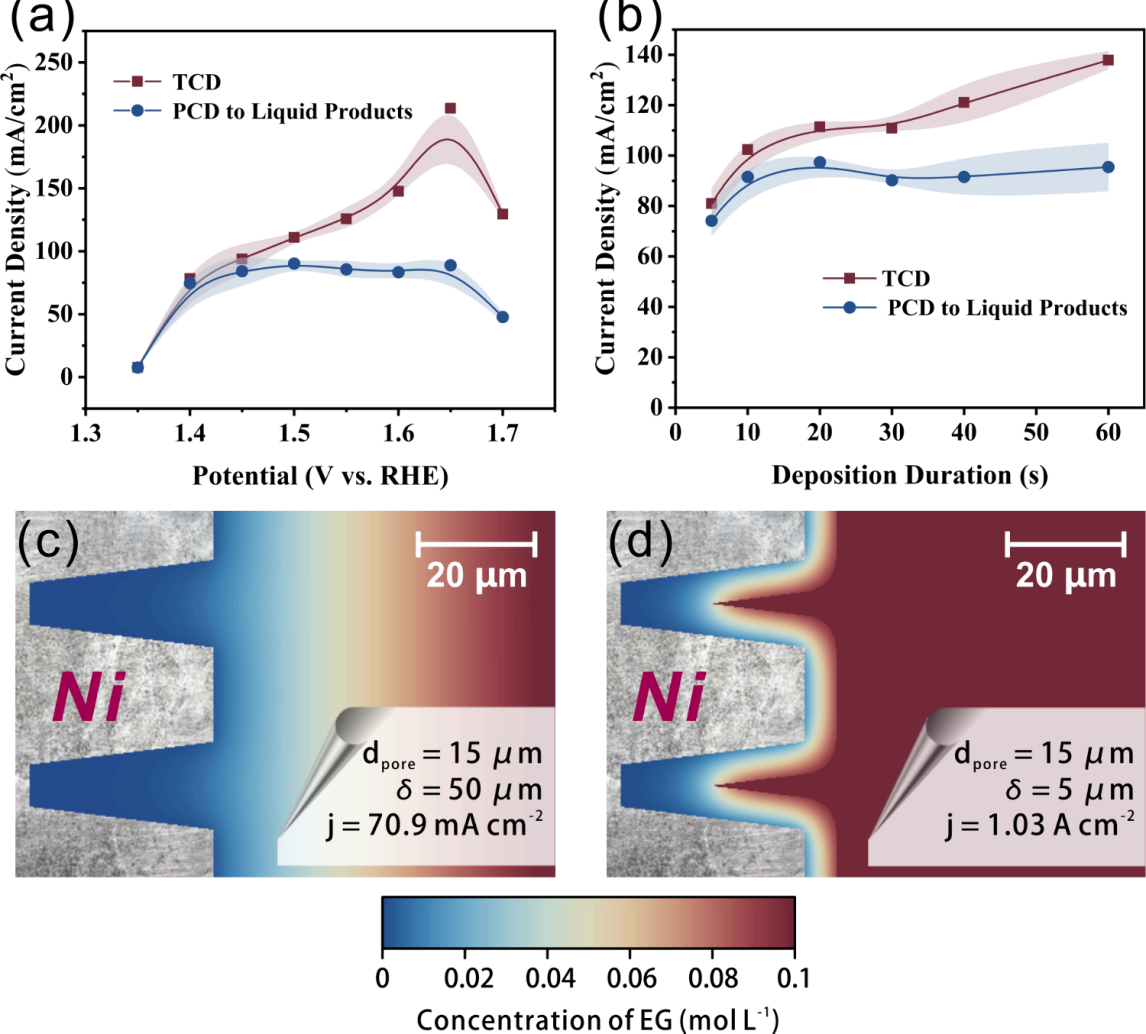
Total current density and partial current density results from H-type cell: (a) potential dependent electrolysis of Ni foam@foil 30s and (b) electrolysis with different deposition durations of Ni foam@foil at 1.5 V vs RHE. Constant charge of 360 C and 100% online *iR*-compensation were applied. (c,d) Calculated reactant (EG) concentration profiles along foam electrode surfaces with different characteristic pore sizes d_pore_, for different values of the diffusion layer thickness δ. In the calculations, 0 mol L^–1^ near-surface and 0.1 mol L^–1^ of bulk concentration was assumed for EG, with a diffusion coefficient of 6.4 · 10^–6^ cm^2^ s^–1^. See the Supporting Information for more details of the calculation and dynamic EG concentration profiles (Figure S36).

To gain more insight into the catalyst utilization and its relevance to the performance metrics, Ni foam electrodes with different thicknesses were analyzed in the H-cell. At 1.5 V vs RHE, the FE of formate declined as the Ni foam layer became ticker (with a longer deposition duration, Figure S33b). A partial current plateau was obtained for electrodes with longer deposition durations than 20 s (Figure 4b), corresponding to the thickness of Ni foam from 15 to 84 μm. This is because EG as the reactant is rapidly consumed inside the 3D Ni foam, due to its high activity and surface area. While ethylene glycol is replenished at the foam surface via mass transport, the deeper regions near the substrate experience reactant insufficiency, as the rate of EG oxidation outpaces diffusion-based replenishment from the bulk electrolyte. This and H_2_O/OH^–^ abundancy shift the current toward OER (Figure S35).

To quantify diffusion-limitations in 3D Ni foam, we implemented a two-dimensional Fick’s law model with a finite diffusion-layer thickness (δ) to map reactant concentrations and predict transport-limited current densities.^48^ Under natural convection (δ = 50 μm), the model yields a limiting current of 70.9 mA cm^–2^ (Figure 4c), which is in agreement with the H-cell plateau (Figure 4a). Notably, foam porosity has minimal impact under these conditions—once the near-electrode layer is depleted, the diffusion front planarizes, and current becomes invariant with pore size (Figure S36). Introducing convective flow (i.e., reducing δ to 5 μm) breaks this plateau, predicting ampere-level currents consistent with experimental data (Figure 4d). Moreover, the open macropores admit the diffusion layer into the foam, activating inner Ni sites and demonstrating strong pore-structure dependence when diffusion is sufficient (Dynamic heat map of EG concentration inside Ni 3D foam for various δ and pore sizes is in Figure S36). These results underscore that alleviating mass-transport constraints is essential to activate the full electroactive surface of foam-structured catalysts and demonstrate the superior performance of 3D porous Ni foam under convective flow. Moreover, our diffusion-layer model explains the systematically lower Faradaic ECSA versus capacitive ECSA (as mentioned before; see Figure S10) by revealing that interior foam regions remain reactant-depleted under diffusion-limited conditions.

### Techno-economic Analysis

A preliminary techno-economic analysis (TEA) was carried out to evaluate the economic feasibility of the integrated electrolysis.^44,49^ Note that the produced formate in KOH electrolyte was assumed to be acidified by additional HCOOH and converted to potassium diformate (KDF), which served as the final product to maximize revenue (Table S3).^18^ The material balance was calculated across three stages: PET hydrolysis, electrolysis, and acidification (Figure S37). With this route, the net profit is estimated up to $807.4 per ton of waste PET (renewable electricity price of 0.06 USD/kWh), with a corresponding payback period of 2.2 years, making it highly competitive in the current market. It demonstrated substantial profit potential despite the price fluctuations of KDF and renewable electricity (Figure S38a). The sensitivity analysis was conducted with an uncertainty range of ± 10% around the base value (Table S4). The sensitivity ranking showed that the KDF price and average FE are the most dominant factors in the process (Figure S38b). These results suggest that although further optimization and large-scale validation are still needed, the integrated process already demonstrates clear economic potential under current conditions.

## Conclusions

In summary, this work demonstrates a paired electrolysis strategy for formate production by integrating CO_2_ reduction (CO_2_RR) with ethylene glycol oxidation (EGOR) derived from PET hydrolysis. Replacing the anodic OER with EGOR resulted in a 51.3% increase in the formate productivity and a 46.3% reduction in energy consumption. Beyond catalyst composition, the study highlights the critical role of electrode structural design, particularly in addressing diffusion limitations at high current densities. The fabricated 3D Ni foam achieved a current density of 1.2 A cm^–2^ for ethylene glycol oxidation to mainly formate, and 100 h stability at 0.5 A cm^–2^ with 93.7% formate FE. The diffusion-enhancing architecture of 3D Ni anode enabled efficient utilization of the active Ni species (NiOOH) within the fluidic electrolyzer. On the cathode side, Bi_2_O_2_CO_3_@GDE modified with PTFE particles demonstrated excellent stability, highlighting the effectiveness of the electrode engineering strategies employed in this study. Furthermore, significant changes in electrolyte composition were identified as a key challenge for long-term operation at ultrahigh current and in the membrane-free electrolyzer, ultimately compromising system stability. Future research will focus on operando monitoring and electrolyte management to sustain high-performance, continuous formate production.

## Supplementary Material





## Data Availability

Data for this article are available at Zenodo at: 10.5281/zenodo.15719073.

## Notes

Experimental procedures, characterization data, and supplementary figures (PDF)
Animation of Figure S36 (AVI)

## Abbreviations

CO_2_: carbon dioxide
PET: Polyethylene terephthalate
GDE: gas diffusion electrode
CO_2_RR: CO_2_ reduction reaction
OER: oxygen evolution reaction
EG: ethylene glycol
EGOR: ethylene glycol oxidation reaction
FE: Faradaic efficiency
BOC: Bi_2_O_2_CO_3_
TPA: terephthalic acid
NO_3_RR: nitrate electroreduction reaction
MPL: microporous layer
CL: catalyst layer
DHBT: dynamic hydrogen bubble templating
ECSA: electrochemical active surface area
DMVCl_2_: dimethyl viologen dichloride
GDL: gas diffusion layer
AEM: anion exchange membrane
CV: cyclic voltammetry
RDE: rotating disk electrode.
