# Supplementary material for: Upcycling CO2 and PET Waste: Ampere-Level Formate Electrosynthesis in an Integrated Electrolyzer
Source: J Am Chem Soc. 2025 Oct 30;147(45):41481–91. doi: 10.1021/jacs.5c11708 (PMC12616681; doi:10.1021/jacs.5c11708)
Supplement: Supplementary file 1 [file ja5c11708_si_001.pdf]

Supporting Information

Upcycling CO_2 and PET Waste: Ampere-level Formate Electrosynthesis in an Integrated Electrolyzer

Xin Yu [a, b], *Hesamoddin Rabiee** [a,b], *Abhijit Dutta* [a, b], *Yaqiang Li* [c], *Zsolt Szakály* [d], *Soma Vesztergom* [d], *Lucas Warmuth* [e], *Alain Rieder* [a, b], *and Peter Broekmann* [a,b]

a. Department of Chemistry, Biochemistry and Pharmaceutical Science, University of Bern, Freiestrasse 3, 3012 Bern, Switzerland. hesamoddin.rabiee@unibe.ch

b. NCCR Catalysis, University of Bern, Freiestrasse 3, Bern, 3012 Switzerland

c. Institute of Molecular Engineering Plus, College of Chemistry, Fuzhou University, Fuzhou 350108, China

d. MTA–ELTE Momentum Interfacial Electrochemistry Research Group, Eötvös Loránd University, H–1117 Budapest, Pázmány Péter sétány 1/A

e. Institute of Catalysis Research and Technology, Karlsruhe Institute of Technology (KIT), Hermann-von-Helmholtz-Platz 1, D-76356 Karlsruhe, Germany

*Corresponding author: hesamoddin.rabiee@unibe.ch

Table of Contents:

1 Experimental **4**

1.1 Synthesis of metal foams (anode) 4

1.2 Fabrication of $Bi_2O_2CO_3$ and GDE (cathode) 4

1.3 Hydrolysis of real-life PET bottle 5

1.4 Structural characterization 5

1.5 Electrochemical characterization 5

1.6 Characterization of electrocatalytic performance 6

1.7 Product analysis 7

1.8 In-situ Raman characterization 8

1.9 Simulation of transport effects 8

1.10 DFT calculations 9

2 Supplementary figures **10**

Figure S1. Top-down SEM, different deposition time (Ni foam) 10

Figure S2. Density and porosity (Ni foam) 10

Figure S3. Ex-situ Raman (Ni foam) 11

Figure S4. XPS (Ni foam) 11

Figure S5. Top-down SEM, different magnification (Ni foam) 12

Figure S6. Cyclic voltammetry (Ni mesh/foil survey) 12

Figure S7. Cross-sectional SEM, thickness, mass loading (Ni foam) 13

Figure S8. Cyclic voltammetry (capacitance, Ni foam) 14

Figure S9. Cyclic voltammetry (viologen, Ni foam) 15

Figure S10. Electrochemically active surface area (Ni foam) 16

Figure S11. Contact angle, static (BOC) 16

Figure S12. Contact angle, time dependence (BOC) 17

Figure S13. Ex-situ Raman and XRD (BOC) 17

Figure S14. Galvanostatic FE 18

Figure S15. Cell voltage of galvanostatic electrolysis 18

Figure S16. FE distributions (stability test) 19

Figure S17. Top-down SEM (post-electrolysis Ni foam) 19

Figure S18. TEM (post-electrolysis Ni foam) 20

Figure S19. Accelerated stability test, FE and cell voltage 20

Figure S20. Top-down SEM and EDX (post-electrolysis BOC) 21

Figure S21. TEM and HRTEM (post-electrolysis BOC) 21

Figure S22. Membrane-free electrolysis, transients 22

Figure S23. Membrane-free electrolysis, yield 22

Figure S24. Linear sweep voltammograms (rotating disc) 23

Figure S25. Tafel analysis 23

Figure S26. Ni species evolution 24

Figure S27. DFT results 24

Figure S28. Top-down SEM of DHBT-deposited Co, Cu, and Fe foams 25

Figure S29. Viologen CVs on DHBT-deposited Co, Cu, and Fe foams 26

Figure S30. EGOR CVs on DHBT-deposited Co, Cu, and Fe foams 26

Figure S31. Electrolysis efficiencies on DHBT-deposited Co, Cu, and Fe foams 27

Figure S32. EG monomer and PET hydrolysate comparison 27
Figure S33. Faradaic efficiency, potentiostatic electrolysis in H-type cell 27
Figure S34. Faradaic and current, EG concentration dependence 28
Figure S35. Illustration of diffusion effects 28
Figure S36. Simulation of diffusion effects 29
Figure S37. Material balance 30
Figure S38. Profitability assumptions and sensitivity ranking 30
3 Performance metrics and comparison to literature 31
Table S1. EGOR performance 31
Table S2. Coupled performance 31
4 Economic feasibility analysis 32
Table S3. Feedstock prices 32
Table S4. Sensitivity analysis 32
Supplementary References 35

Experimental

1.1 *Synthesis of metal foams (anode)*

Ni foam was electro-deposited according to the dynamic hydrogen bubble template (DHBT) method (Scheme 1), in which 0.12 M $NiSO_4$ (Sigma-Aldrich, ≥ 98%) and 1.5 M NH_4Cl (Sigma-Aldrich, ≥ 99.5%) were applied as the bath solution. Ni foil (0.25 mm thick, Alfa Aesar, 99.5% metal basis) and Ni wired mesh (4N, d_{wire} = 0.2 mm, d_{pore} = 0.3 mm, HaiFu, China) as the substrate was successively ultrasonicated in isopropanol (Sigma-Aldrich, ≥ 99.0%) and 0.1 M HCl (Sigma-Aldrich, 35-37%) for 30 min, prior to electrodeposition. The deposition was performed on Metrohm multi Autolab M204 (NOVA 2.1 software, with a 10 A booster) with −3 A cm^{-2} current density for 5, 10, 20, 30, 40 and 60 s, respectively. As-deposited Ni foam was rinsed with Milli-Q water (18.2 MΩ cm and TOC value below 5 ppb, Millipore) thoroughly and dried overnight at room temperature.

Other metal foams (Co, Fe, and Cu) were prepared by the DHBT method using identical Ni foils as substrates. The corresponding deposition baths were composed of 1.5 M NH_4Cl + 0.1 M $CoSO_4$ + 0.01 M sodium citrate monobasic for Co, $(NH_4)_2SO_4$ + 0.1 M $FeSO_4$ + 0.05 M sodium citrate monobasic for Fe, and 1.5 M H_2SO_4 + 0.2 M $CuSO_4$ for Cu. Electrodeposition was performed at a current density of –3 A cm^{-2}, with deposition times of 15 s, 90 s, and 10 s for Co, Fe, and Cu foams, respectively. All reagents (analytical grade, Sigma-Aldrich) were used as received without further purification.

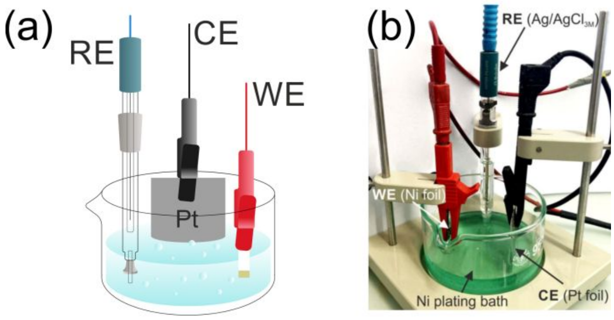

Scheme S1. (a) Schematic and (b) experimental set-up for the electrodeposition of Ni and other metal foam catalysts.

1.2 *Fabrication of $Bi_2O_2CO_3$ and gas diffusion electrode (GDE, cathode)*

Precipitation method for $Bi_2O_2CO_3$ catalyst (powder): The synthesis of $Bi_2O_2CO_3$ was carried out by suspending 234 mg of Bi_2O_3 (Sigma-Aldrich, 99.8% trace metals basis) in 10 ml of Milli-Q water and dissolving it by stirring after addition of 3 mL concentrated HNO_3 (Sigma-Aldrich, ACS grade, 68.0-70.0%). Precipitation was carried out using 2.5 g of Na_2CO_3 (ACS grade, Sigma-Aldrich) dissolved in 10 mL of Milli-Q water. This solution was then added to the Bi^{3+}-containing one until pH 7 was reached. Afterwards, suspension ageing took place at 85 °C for 3 h to enforce crystallization of $Bi_2O_2CO_3$. Finally, the white product was filtered off, washed five times with 20 mL Milli-Q water and dried at 70 °C for 12 h.

Fabrication of $Bi_2O_2CO_3$-based GDE: The catalyst ink was prepared according to the following recipe: 100 mg as-prepared $Bi_2O_2CO_3$, 50 mg PTFE micro-particles (polytetrafluoroethylene, powder, 1 µm particle size, Sigma-Aldrich) and 0.11 mL Nafion 117 perfluorinated resin solution (w_{Nafion} = 5%, Sigma–Aldrich), mixed and ultra-sonicated in 20 mL iso-propanol for 30 minutes. Then the ink was air-

brushed on a GDL (Gas diffusion layer, 6.3 cm × 6.3 cm, Sigracet 39BB) until the mass loading reached 1.5 mg cm^{-2}, as illustrated in Scheme 2.

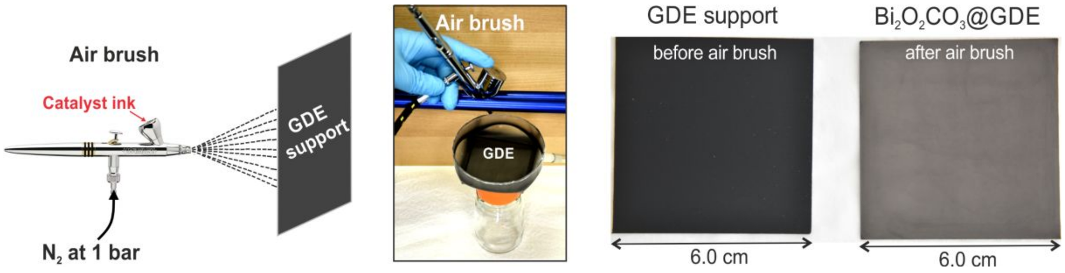

Scheme S2. Schematic illustration of air-brushing process for $Bi_2O_2CO_3$@GDE.

1.3 *Hydrolysis of real-life PET bottle*

A real-life PET bottle was thoroughly cleaned, dried, and cut into small pieces (5–10 mm). The PET pieces were then hydrolyzed in 80 mL of 3.0 M KOH solution (ACS grade, Sigma-Aldrich) at 95 °C on a hot plate with vigorous magnetic stirring. After 24 h of reaction, 1.33 g of PET was hydrolyzed, and the resulting hydrolysate was collected directly for subsequent electrolysis.

1.4 *Structural characterization*

The structural analysis of the electrode morphologies was conducted before and after electrolysis through scanning electron microscopy (SEM) with a Zeiss Gemini 450 instrument equipped with an InLens secondary electron and a backscattered electron detector. AZtec 4.2 software (Oxford Instruments) was applied to acquire energy dispersive X-ray (EDX) point spectra and the respective 2D elemental mappings. X-ray powder diffractograms were recorded with a Bruker D8 diffractometer with CuKα radiation (λ = 0.1540 nm, I = 40 mA) generated at 40 keV acceleration voltage. Diffractograms were measured in the 2θ range from 10° to 90°, with a scan rate of 1° min^{-1}. The ex-situ Raman spectra were acquired by a LabRAM HR800 confocal microscope (Horiba Jobin Yvon), with a laser of 633 nm wavelength, laser power of 3 mW, calibrated with a standard silicon wafer (520.6 cm^{-1}). XPS measurements were carried out using a PHI VersaProbeII scanning XPS micro-probe (Physical Instruments AG, Germany) equipped with a monochromatic Al Kα X-ray source operated at 24.8 W with a spot size of 100 µm. The spherical capacitor analyzer was set at 45° take-off angle with respect to the sample surface. The pass energy was 46.95 eV. Peak positions were referenced to the carbon C1s peak at 284.8 eV. The curve fitting was performed using the Avantage and XPS peak software. For HR-TEM imaging, a FEI Tecnai G2 F20 instrument equipped with a thermal (Schottky) field-emission source was used and operated at a 200 kV accelerating voltage. The microscope objective lens was a FEI Tecnai "Twin" lens type. A spherical aberration coefficient of Cs = 2.2 mm permitted a point resolution of 2.7 Å and a line resolution of 1.44 Å. The images were taken on a CETA CCD camera.

1.5 *Electrochemical characterization*

All the electrochemical measurements (unless otherwise specified) were carried out in an H-type cell with 3-electrodes configuration, taking Ag/AgCl (saturated KCl) as the reference electrode (RE, Metrohm), Pt foil as the counter electrode (CE). All potential values reported in the paper were converted to reversible hydrogen electrode (RHE) according to the Nernst equation:

$$E_{\mathrm{RHE}} = 0.198\ \mathrm{V} + 0.0592\ \mathrm{V} \times p\mathrm{H} + E_{\mathrm{measured}}. \quad (1)$$

Cyclic voltammetry (CV) and linear scan voltammetry (LSV) were conducted with a scan rate of 10 mV s^{-1}, taking 1 M KOH as supporting electrolyte. The result curves were 85% iR-compensated

offline, wherein the resistance value was obtained from electrochemical impedance spectroscopy (EIS). As for the EIS measurement, 5 mV amplitude was applied at open circuit potential (OCP) in a frequency range from 10^5 Hz to 1 Hz. In addition, electrochemical active surface area (ECSA) was measured through cyclic voltammetry with a dimethyl viologen redox-active probe ($DMVCl_2$, Sigma-Aldrich, 98%). The CV curves were collected in 10 mM $DMVCl_2$ and 0.5 M Na_2SO_4 (Sigma-Aldrich, ≥ 99.0%), with the scan rate of 20-140 mV s^{-1}. Only the last one was taken from the 15 scans at each scan rate, being used for the calculation according to the Randles–Ševčík equation, Equation (2):

$$i_p = 0.4463\, nFAc\sqrt{\frac{nFvD}{RT}} \quad (2)$$

where i_p is the peak current in the CV curves, n is number of transferred electrons for the reaction, $F = 96485.3$ C mol^{-1} is the Faraday constant, A is the surface area of the electrode, c is the reactant concentration in the bulk solution, D is the diffusion coefficient, v is the scan rate, R is the gas constant, T is the temperature.

Rotating disc electrode system (RDE, PINE rotator unit) was used for analysis, with a Ni disc working electrode (diameter: 5 mm), Pt foil counter electrode and Ag/AgCl (saturated KCl) as the reference electrode in a single cell. LSV measurements with 1.0 M KOH supporting electrolyte, different concentrations of EG (Sigma-Aldrich, ≥ 99%), scan rate of 1 mV s^{-1}, rotating range of 400–1600 rpm were implemented. The data points were collected to fit the Tafel slope, the parameter b in Equation (3):

$$\eta = a + b\log j \quad (3)$$

where η is the overpotential of the target reaction, a is the exchange current density, b is the Tafel slope, j is the current density. Also, 100% iR-correction was applied to the LSV curves before getting into Tafel fitting.

1.6 Characterization of electrocatalytic performance

The electrocatalytic performance of Ni foam was evaluated in H-type cell and flow cell, respectively. First, the configuration of H-type cell is same as above, with two compartments separated by an anion exchange membrane (AEM, Fumasep FAA-3-PK-130). The experiments were carried out under potentiostatic conditions, with constant charge of 360 C. Note that 100% online iR compensation was applied with Autolab PGSTAT128N, wherein 5 mV amplitude was applied at a frequency of 1kHz during the elelctrolysis.

EG oxidation reactions taking place at the anode are given in Reactions (R1) and (R2),

$$C_2H_6O_2 + 8OH^- \rightarrow 2HCOO^- + 6H_2O + 6e^-, \quad (R1)$$

$$C_2H_6O_2 + 5OH^- \rightarrow C_2H_3O_3^-(\text{glycolate, GA}) + 4H_2O + 4e^-, \quad (R2)$$

while the CO_2RR reactions taking place at the cathode are

$$CO_2 + H_2O + 2e^- \rightarrow HCOO^- + OH^- \quad (R3)$$

and

$$CO_2 + H_2O + 2e^- \rightarrow CO + 2OH^-. \quad (R4)$$

Assuming that formate is the majority product on both electrodes, the overall cell reaction can be written as

$$C_2H_6O_2 + 3CO_2 + 5OH^- \rightarrow 5HCOO^- + 3H_2O. \quad \text{(R5)}$$

For all electrolysis, the total average current density (*TCD*) was calculated as

$$TCD = \frac{I}{A}, \quad (4)$$

where *A* denotes the (geometric) surface area of the electrode and *I* the averaged current. The latter is estimated as

$$I = \frac{Q_{\text{total}}}{t}, \quad (5)$$

that is, as a ratio of the total electrolysis charge Q_{total} and the *t* electrolysis time.

Based on Reactions (R1)–(R5), the Faradaic efficiency (*FE*) of liquid and gaseous products was calculated either as:

$$FE_{\text{liquid, i}} = \frac{n \cdot F \cdot c_i \cdot V}{Q_{\text{total}}} \times 100\%, \quad (6)$$

where c_i denotes the concentration of the ith liquid product after electrolysis and *V* the volume of electrolyte, or as

$$FE_{\text{gas, i}} = \frac{x_i \cdot F \cdot n \cdot v}{V_{\text{m}} \cdot I} \times 100\%, \quad (7)$$

where x_{i} denotes the mole fraction of the product in the outflow gas, *v* denotes the volumetric gas flow rate, and $V_{\text{m}} = 24.4$ L mol^{-1} is the volume constant of gas at room temperature and pressure.

The partial current density (*PCD*) corresponding to a given product was then calculated as

$$PCD_i = TCD_i \times FE. \quad (8)$$

In the flow cell, we applied a two-electrode configuration for the co-production of formate, in which Ni foam deposited on Ni mesh acting as the anode, and $(BiO)_2CO_3$ GDE as the cathode. With this configuration, different currents in the range of 0.2 to 1.2 A were applied and stability tests were conducted at 0.5 A and 1.0 A, respectively. The reactions from both anode and cathode sides are listed above (R1-R6). Besides, the total *FE* of cathode was calibrated to 100% because of the formate cross-over through the AEM. Formate productivity and energy consumption were determined by Equations (9) and (10):

$$\text{Formate productivity} = \frac{c_{\text{formate}} \times V}{t}, \quad (9)$$

$$\text{Energy consumption} = \frac{I \times U}{\text{Formate productivity}}, \quad (10)$$

where *U* is the time-averaged cell voltage.

1.7 Product analysis

Ion-exchange chromatography (IC) was applied to quantify concentrations of formate and aqueous by-products from EGOR and CO_2RR. The analysis was performed on Metrohm 940 Professional IC Vario with MagIC Net 3.3 program, where 3 mmol L^{-1} Na_2CO_3 and 0.1 mol L^{-1} H_2SO_4 served as the

eluent and the suppressor, respectively. For the calibration of the IC instrument, known standard formate/glycolate concentration solutions were prepared in the range of 1 to 100 ppm by dilution of 1000 ppm IC standard solutions (Sigma–Aldrich). Online gas chromatography (GC, GC 8610C, SRI Instruments), equipped with a thermal conductivity detector (TCD) and a flame ionization detector (FID), was connected to the gas outlet of the flow cell to analyze the concentrations of H_2 and CO. Standard mixed gas was applied to the calibration. Nuclear magnetic resonance (NMR, Bruker Avance II 400 spectrometer) was appointed to analyze the products of PET hydrolysis and electrolysis, in which the recipe of samples was 5 μL DMSO (internal standard), 100 μL D_2O and 500 μL solution to be tested.

1.8 In-situ Raman characterization

In-situ Raman was performed to investigate the in-situ evolution of nickel catalyst, in a single cell equipped with 3-electrodes configuration, Ni foam was deposited on 3mm diameter Ni disc served as the working, an Au wire as the counter, Ag/AgCl (saturated KCl) as the reference electrode. The Raman spectra were acquired by LabRAM HR800 confocal microscope (Horiba Jobin Yvon) operated by LabSpec 3.0 softwere, with a He-Ne laser of 633 nm wavelength, laser power of 3 mW, being calibrated with a standard silicon wafer (520.6 cm^{-1}). A long working distance objective with 50x magnification and 8 mm focal length was used to focus the laser. The data acquisition was executed 10 times (10 s each) in the range of 200-800 cm^{-1}, being displayed after averaging. The Raman peaks were deconvolved and integrated according to the Lorentz model. The measurement was conducted in 1 M KOH (w/ or w/o 100 mM EG), in multiple steps from OCP (ca. 0.6V) to 1.6 V vs. RHE, with a stabilization time of 60 s between each step.

1.9 Simulation of transport effects

To ensure maximum catalyst utilization, it is necessary that the 3D electrode has sufficient contact with reactant in the electrolyte. The achievable current density, normalized to geometric surface area, can be effectively increased if we create foams with relatively large pore sizes, and at the same time apply an electrolyte flow that is able to push the diffusion front into the pores. The simulated reactant concentration profiles and current densities of Fig. 4 in the main text demonstrated this effect by a simple numerical solution of Fick's diffusion equations in two dimensions.

The effect of varying the pore size d_{pore} (which is set either to 3 or 15 μm) and the diffusion layer thickness δ (δ = 50 μm corresponds to the case of natural, 5 μm to that of forced convection) is further illustrated here in the animated Figure S35. For these simulations, a home-built program was written that utilized a Cartesian grid with 200 nm cell size. At the beginning of the simulation, each (solution containing) cell was set to contain ethylene glycol (EG) in a 0.1 M concentration. In each simulation step, the concentration of the near-surface cells was zeroed (thus contributing to the simulated current), and the discretized version of Fick's diffusion equation (with a diffusion coefficient of $6.4 \cdot 10^{-6}$ cm^2 s^{-1}) was solved over the simulation grid to propagate the effect of near-surface concentration changes. Finally, in order to model the effect of convection, the concentration of cells that lied out of a δ Euclidean distance from the surface was set to the bulk concentration (0.1 M). In order to simulate the diffusion process, a fixed time step of Δt = 1 ms was used and new concentrations over the grid were calculated from the previous ones by using a truncated Gaussian filter approximation of the exponential of the discrete Laplace operator.[1] Current values were calculated by summing the amount of substance in the zeroed-out (near-electrode) cells, dividing these sums by the Δt time-step, and scaling by n F (where n = 6 is the number of electrons consumed in EGOR and F is Faraday's constant).

1.10 DFT calculations

DFT calculations were carried out by Vienna ab initio Simulation Package (VASP).[2] Projector augmented wave (PAW)[3] was accepted to describe the interaction among electrons and nucleus. The generalized gradient approximation parameterized by Perdew, Burke, and Ernzerhof was used to solve the exchange-correlation function.[4] DFT-D3 method of Grimme with zero-damping function was adopted to describe the van der Waals (vdWs) effects. The NiOOH catalyst with monoclinic structure was established along (010) crystal plane. The simulation box was 14 Å and 16 Å in length and width, respectively, with the vacuum layer thickness along the c direction for the simulation box set as 20 Å to avoid the periodic interactions. Monkhorst-Pack k point was used with k point set as $4 \times 4 \times 1$. The cutoff energy of the plane-wave basis was set to 500 eV.[5] During optimization calculation, the bottom atomic layer was fixed while the top three atomic layers were fully relaxed until the maximum force on each atom was less than 0.05 eV $Å^{-1}$. and the convergence criterion of the self-consistent field method was set to 10^{-5} eV between two electronic steps. Spin polarization was considered for all calculations.

The Gibbs free energy (ΔG) was obtained according to computational hydrogen electrode (CHE) model[6,7] proposed by Nørskov and co-workers as follows:

$$G = E + E_{ZPE} - TS \tag{11}$$

where E is the calculated energy, E_{ZPE} represents the zero-point vibration energy, T (298.15 K) is temperature and S is entropy. The zero-point vibration energies and entropies for adsorbates were calculated from the vibration frequencies, while the Gibbs free energies for single molecules were taken from standard tables.[8]

Supplementary figures

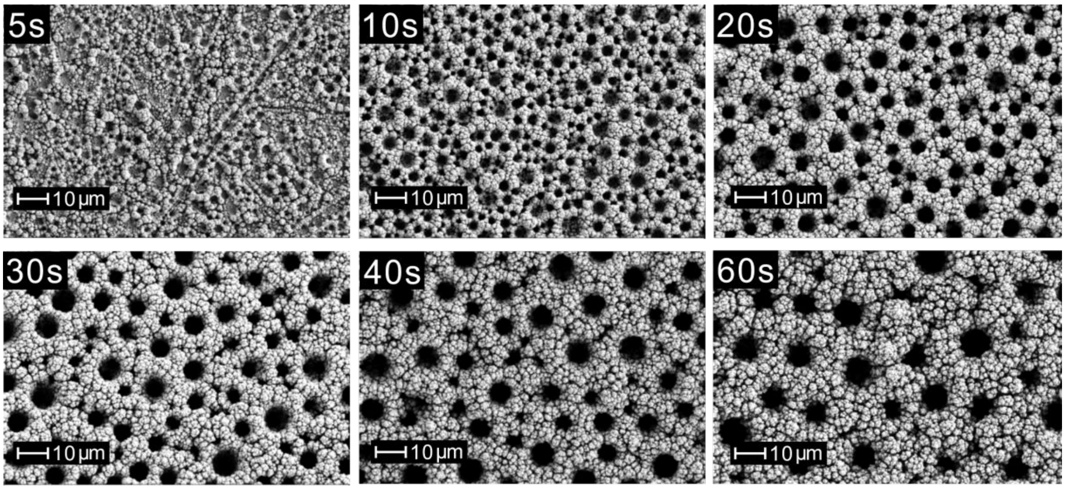

Figure S1. Top-down SEM images of Ni foam deposited on Ni foil in different deposition durations.

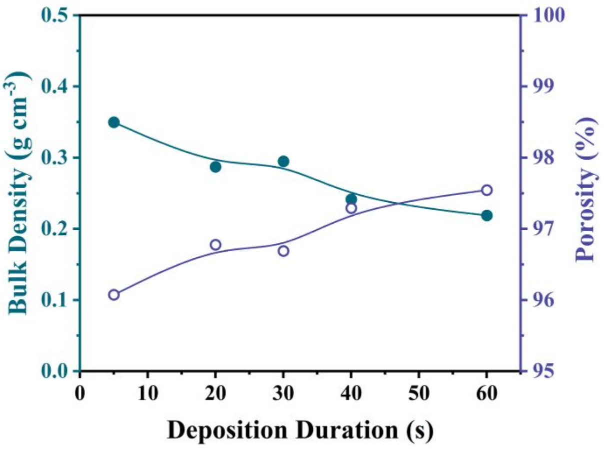

Figure S2. Bulk density and porosity of Ni foam with different deposition durations. Bulk density is calculated based on the mass loading and geometric volume of Ni foam layer, which is further compared to conventional Ni density (8.902g cm^{-3}) to get the porosity of as-deposited Ni foam.

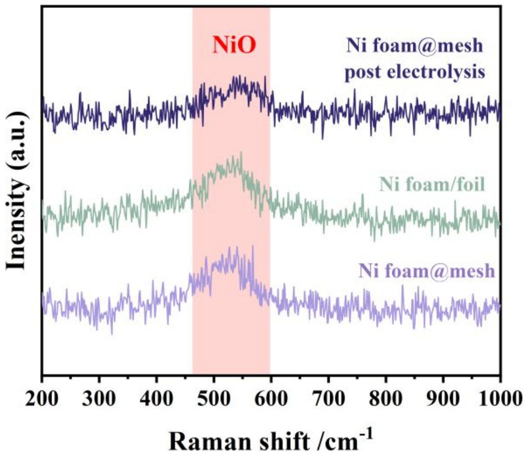

Figure S3. *Ex-situ* Raman spectra on Ni foam before and after electrolysis. NiO is identified at 540 cm^{-1} on all samples, indicating the oxide shell/nanoflakes on Ni foam.

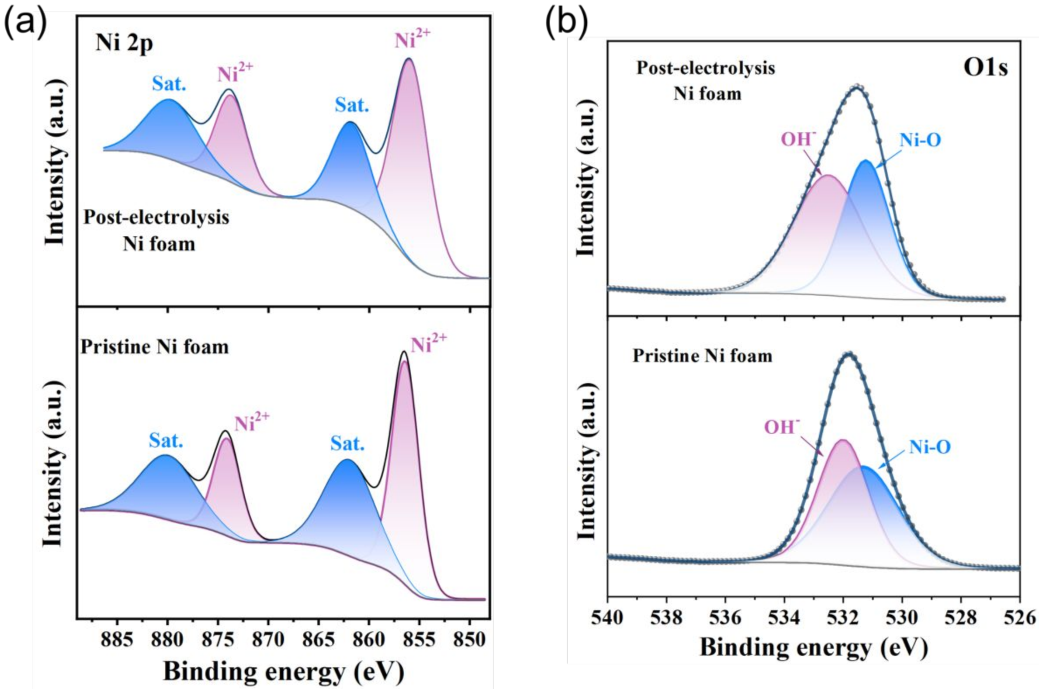

Figure S4. XPS spectra of (a) Ni 2p and (b) O1s obtained from pristine and post-electrolysis (100 h at 500 mA) Ni foam.

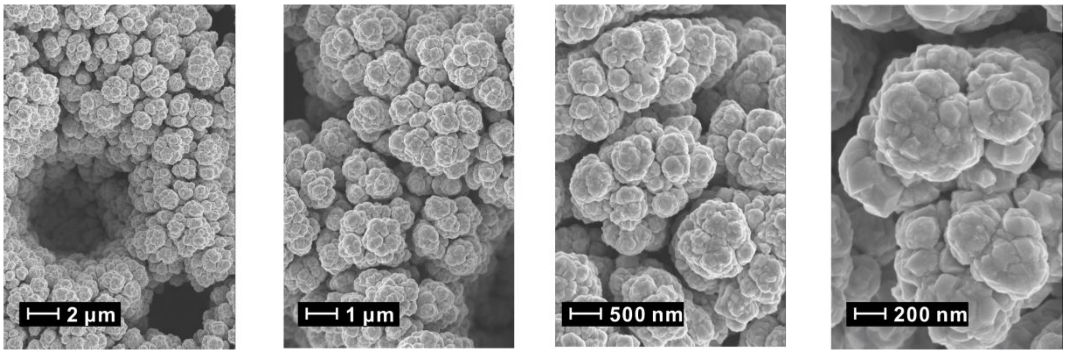

Figure S5. Top-down SEM images of Ni foam 30s deposited on Ni foil in different magnifications.

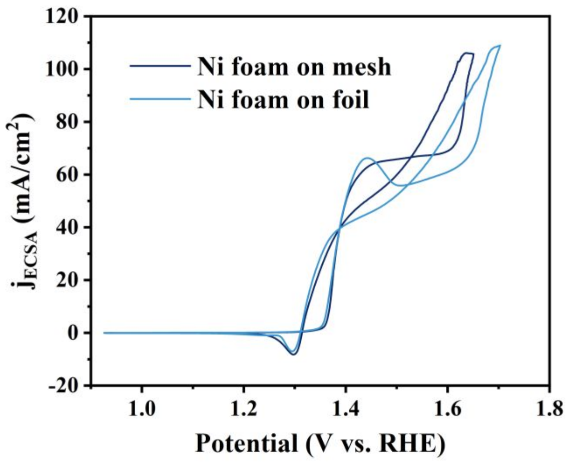

Figure S6. Cyclic voltammetry curves of Ni foam 30s deposited on Ni foil and Ni mesh, with the condition of 1 M KOH + 100 mM EG, 10 mV/s sweep rate in H-type cell. The current densities were normalized by the ECSA (redox method).

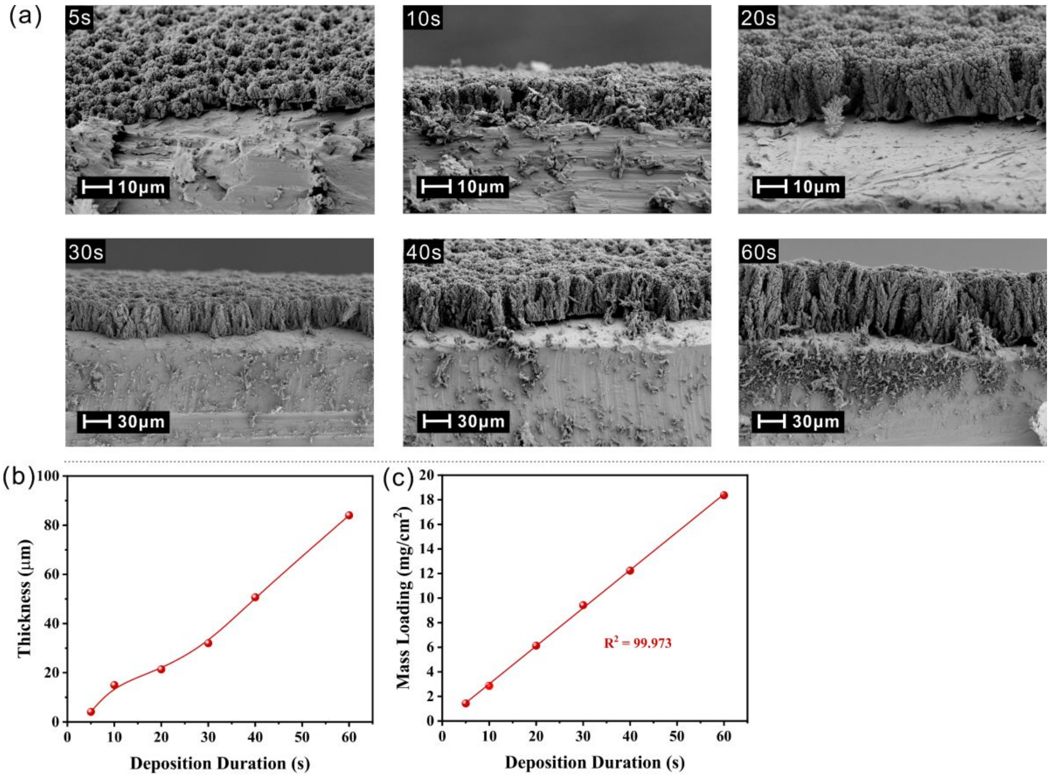

Figure S7. (a) SEM images for the cross-section, (b) thickness, and (c) mass loading of Ni catalyst on Ni foam@foil with different deposition duration. The mass loading exhibits a proportional relationship with the deposition time, indicating a highly controllable deposition process.

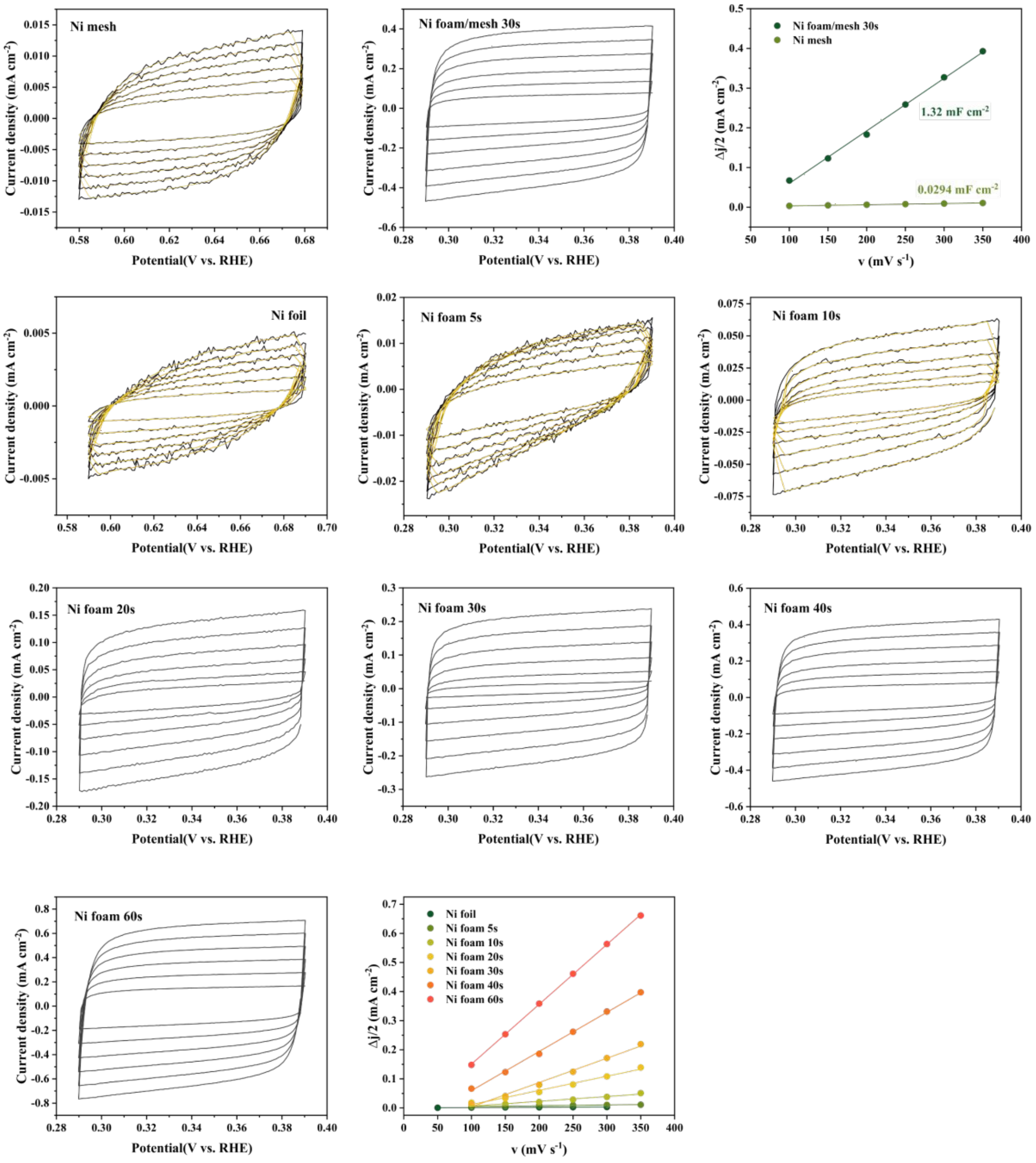

Figure S8. Cyclic voltammetry curves of Ni foam and corresponding electrochemical active surface area (ECSA) determined by capacitance method. 100 mV potential window around OCP (open circuit potential) was chosen with 0.5 M Na_2SO_4 as the electrolyte, the scan rates of 100 to 350 mV s^{-1}. Only the last cycle was presented as the result among the 20 scans at each scan rate.

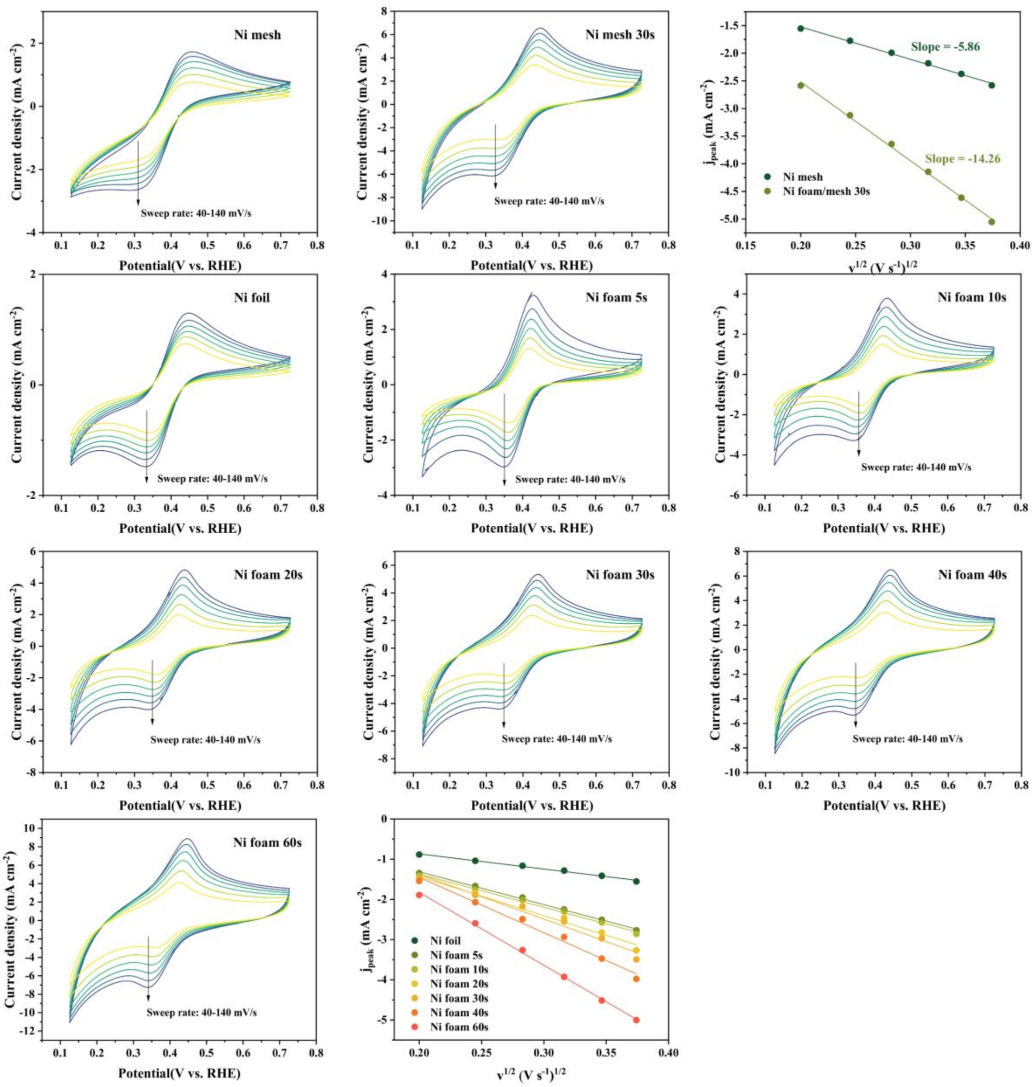

Figure S9. Cyclic voltammetry curves of Ni foam and corresponding ECSA determined by $DMVCl_2$ redox. The CV curves were collected in 10 mM viologen + 0.5 M Na_2SO_4, with the scan rates of 20–140 mV s^{-1}. Only the last one was taken from the 15 scans at each scan rate, being used for the calculation according to the Randles–Ševčík equation, Equation (1).

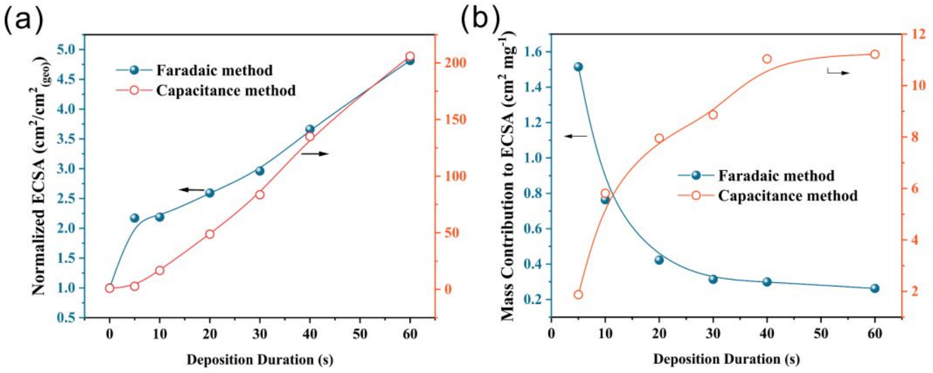

Figure S10. (a) Normalized electrochemistry active surface area (ECSA) and (b) mass contribution to ECSA determined by capacitive and Faradaic ($DMVCl_2$ redox) methods

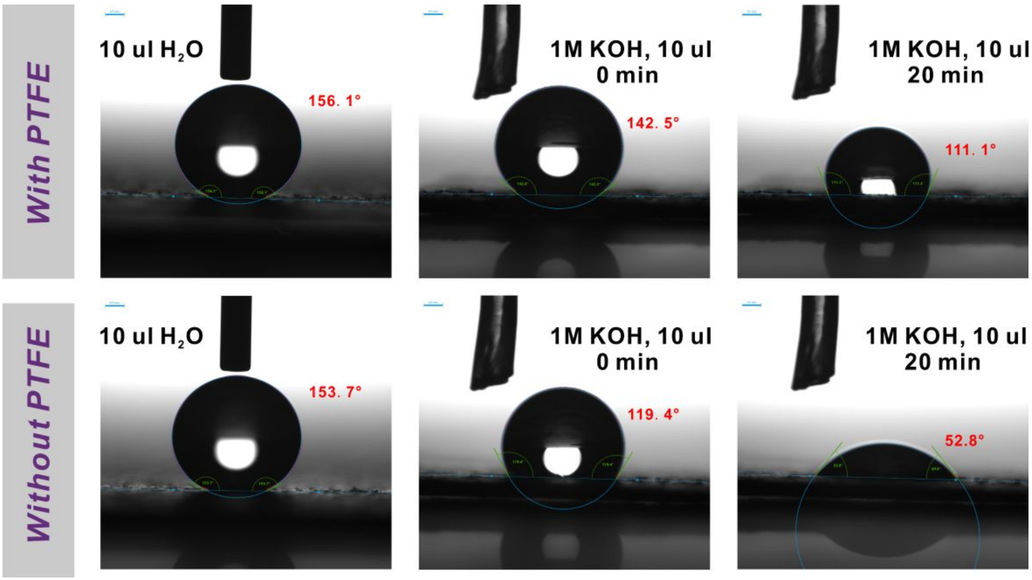

Figure S11. Contact angle measurements of BOC@GDE: effect of PTFE doping. Drops with Milli-Q water and 1.0 M KOH were tested on the GDEs. Angle changes in the KOH case were recorded in a duration of 20 min.

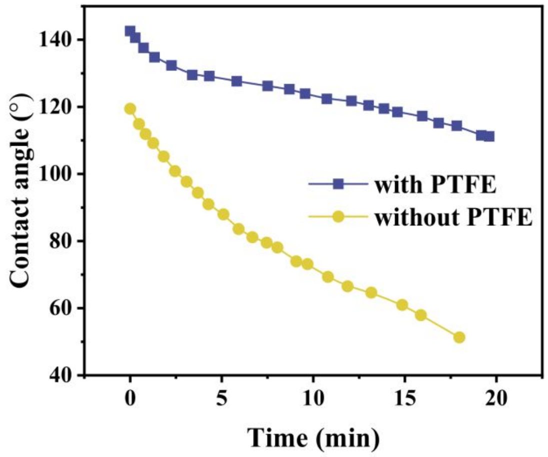

Figure S12. Time-dependence of contact angle of BOC@GDE with 1.0 M KOH.

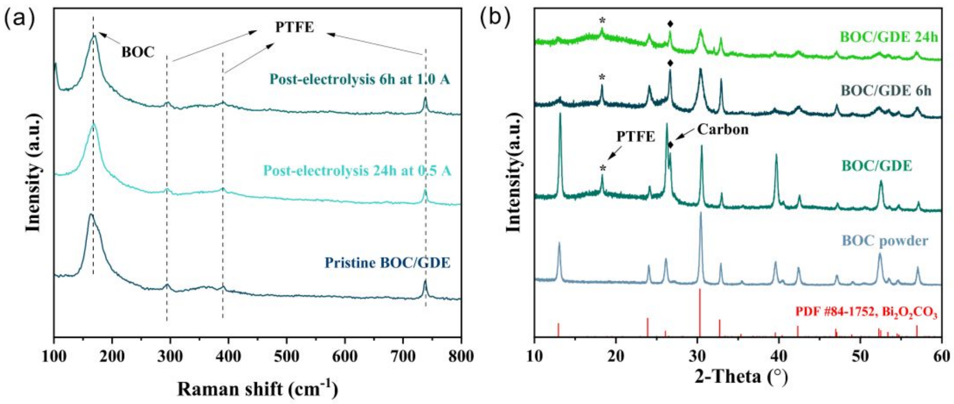

Figure S13. (a) *Ex-situ* Raman spectra and (b) XRD patterns of $Bi_2O_2CO_3$ on the GDE (before and post electrolysis in the flow cell).

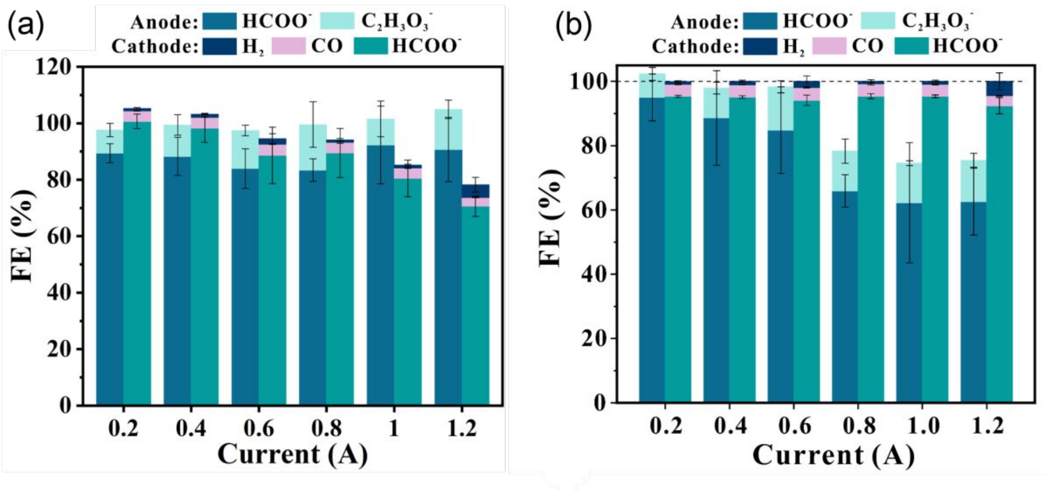

Figure S14. (a) Original and (b) corrected FE results of galvanostatic electrolysis from the EGOR+CO_2RR design, with 1.0 M KOH as catholyte and 1.0 M KOH + 1.0 M EG as anolyte. The original total FE from cathode is less than 100%, due to the formate cross-over through the anion exchange membrane (AEM). It was then corrected to 100% based on the integrated formate yield to quantify the anodic FE.

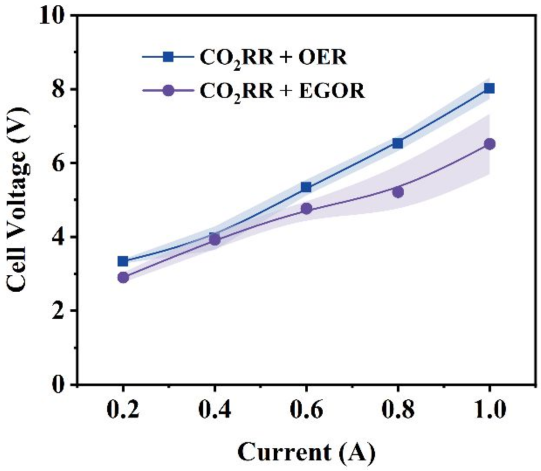

Figure S15. Cell voltage of galvanostatic electrolysis in the flow cell of CO_2RR integrated by OER and EGOR, with 1.0 M KOH as the supporting electrolyte for both sides and 1.0 M EG added for EGOR on the anode.

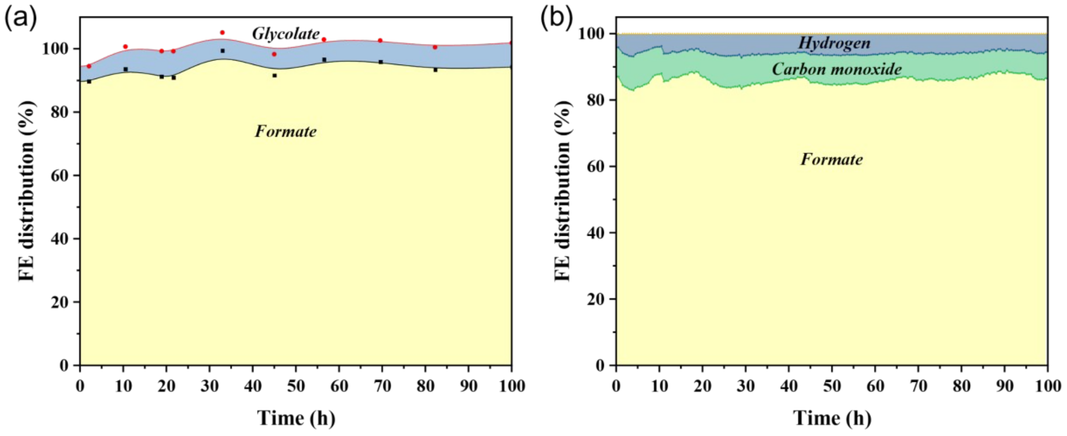

Figure S16. FE distributions of (a) EGOR and (b) CO_2RR during the stability test, which was conducted in the integrated cell at 500 mA for 100 hours.

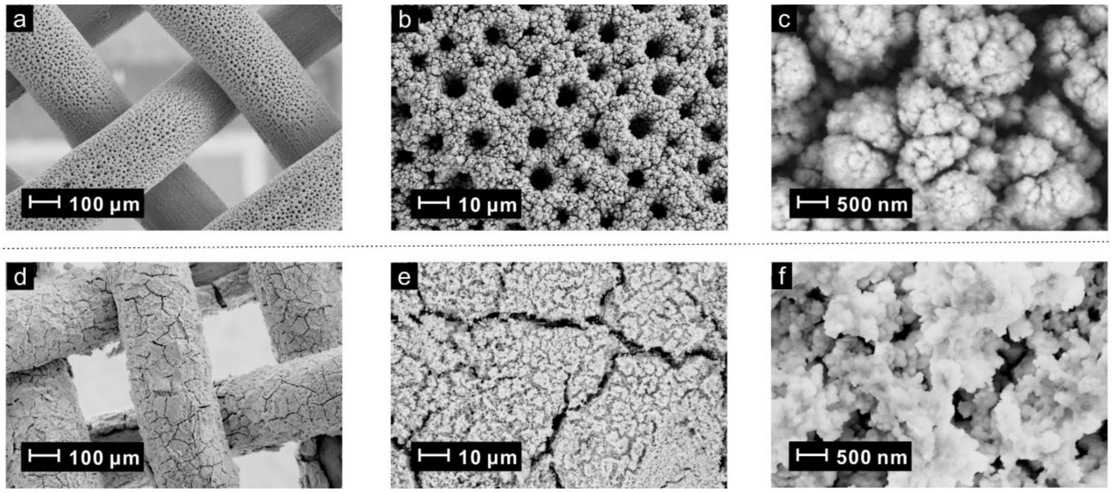

Figure S17. Top-down SEM images of Ni foam after long-term electrolysis: (a-c) 100 h at 500 mA and (d-f) 6 h at 1.0 A

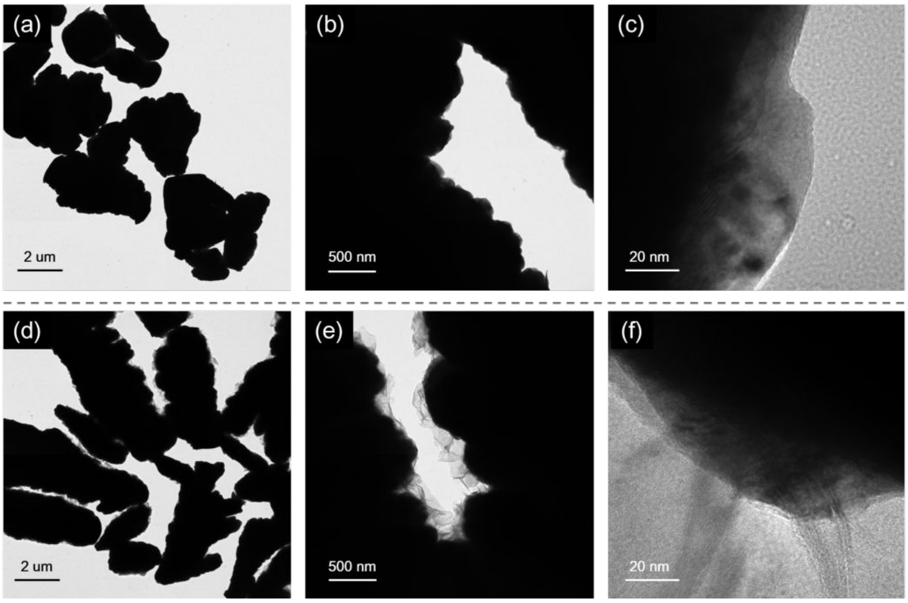

Figure S18. TEM images in different magnifications of (a-c) pristine and (d-f) post-electrolysis (100 h at 500 mA) Ni foam. The Ni particles were detached from the foam electrode via ultrasonication.

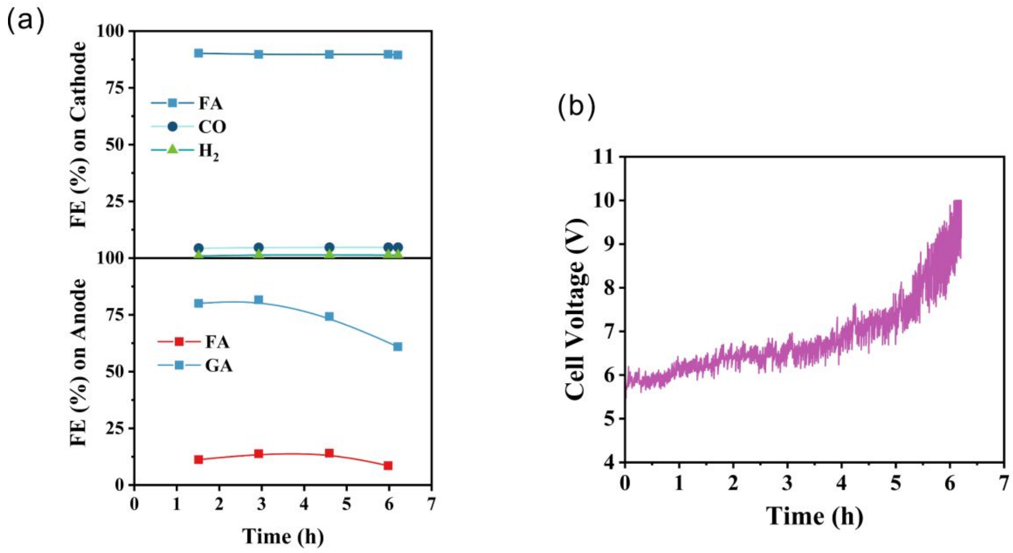

Figure S19. (a) FE and (b) cell voltage of the accelerated stability test at 1.0 A for the CO_2RR and EGOR integrated system.

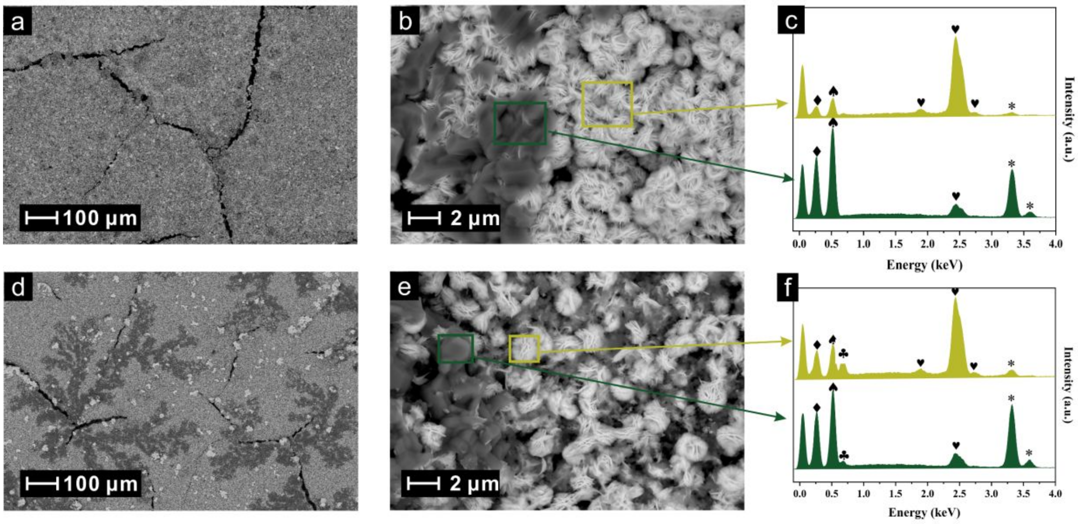

Figure S20. SEM images and EDX spectra of $Bi_2O_2CO_3$ GDE, which are (a-c) after 6 h at 1.0 A, and (d-f) after 100 h at 500 mA, respectively. In the EDX spectra (c) and (f), ♦ refers to carbon, ♠ to oxygen, ♣ to fluorine, ♥ to bismuth, and ✻ to potassium, respectively.

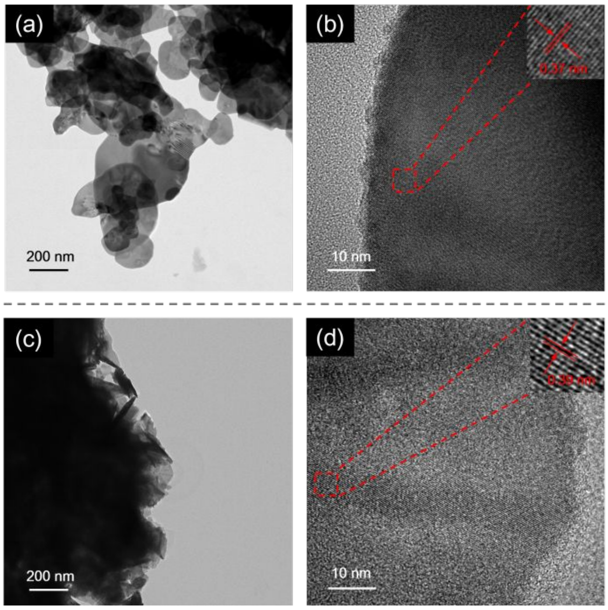

Figure S21. TEM and HRTEM images of (a-b) pristine and (c-d) post-electrolysis (100 h at 500 mA) $Bi_2O_2CO_3$ catalyst. The latter one was stripped off the corresponding GDE.

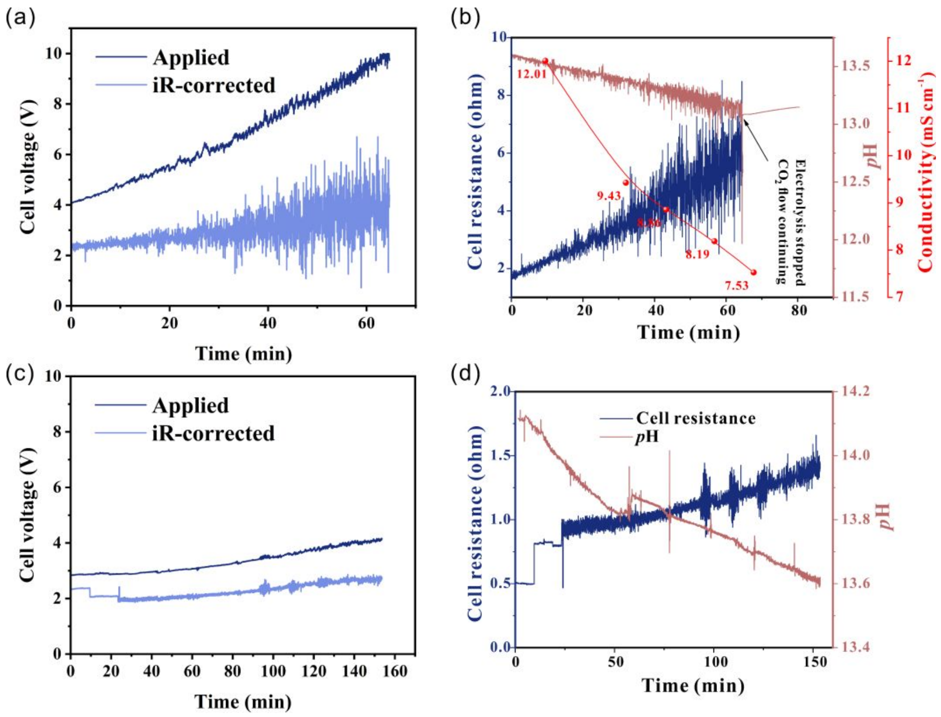

Figure S22. Electrolysis data from the membrane-free flow cell at 1.0 A: (a) cell voltage and (b) electrolyte information in 1M KOH+1M EG; (c) cell voltage and (d) electrolyte information in 3M KOH+1M EG. The resistance value for iR correction was obtained from an online high frequency impedance. The pH value was acquired from a pH meter inserted in the electrolyte tank. Conductivity was measured by a conductivity meter from electrolyte sampling.

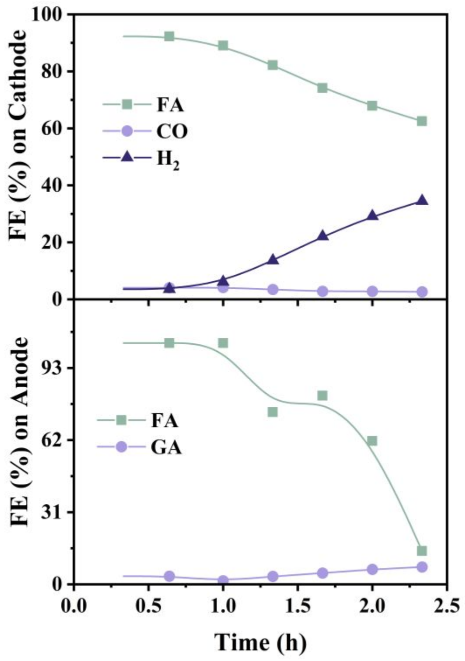

Figure S23. Faradaic efficiency in the AEM-free electrolyzer with 3M KOH+1M EG, at the current of 1.0 A, where the total FE of cathode was compensated to 100% to quantify the anodic FE.

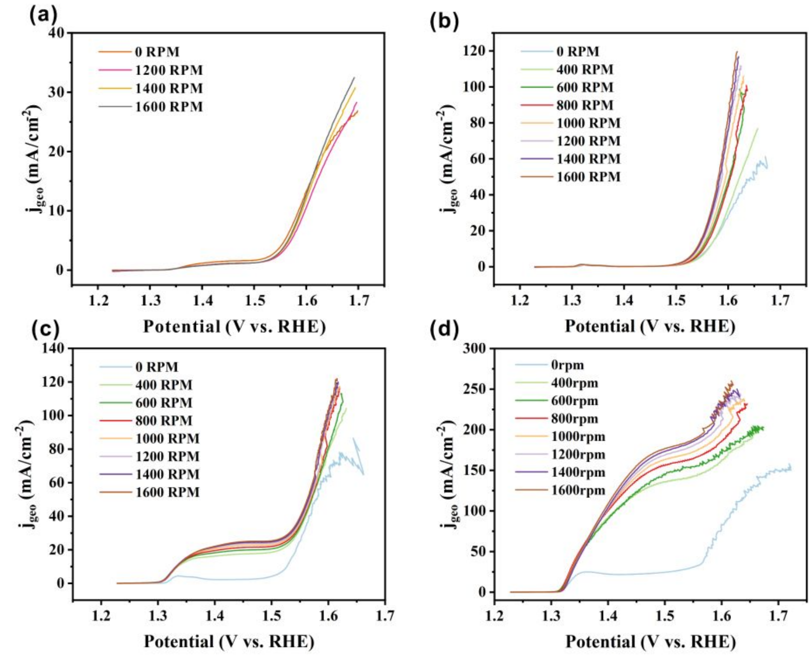

Figure S24. LSV curves acquired from a RDE configuration, in which the Ni foam was deposited on a Ni disc (d = 5mm) for 30 s at the current density of -3 A cm^{-2}, Pt wire as counter electrode. (a) Ni disc in 10 mM EG, Ni foam in (b) blank KOH, (c) 10 mM EG, and (d) 100 mM EG. Scan rate of 1 mV s^{-1} and 100% iR compensation were applied to exclude capacitance and resistance effect.

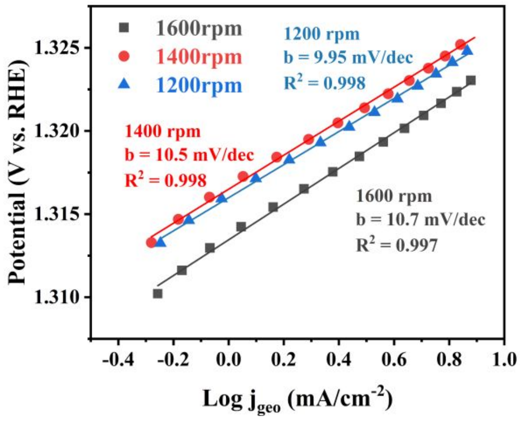

Figure S25. Tafel fitting results of Ni foam@disc in 100 mM EG+1.0 M KOH at different rotating rates in the RDE system. The deviation from 1200 to 1600 rpm is negligible, indicating the effect of diffusion was successfully mitigated to guarantee the neat kinetic current. The slope at 1400 rpm was taken as the final result.

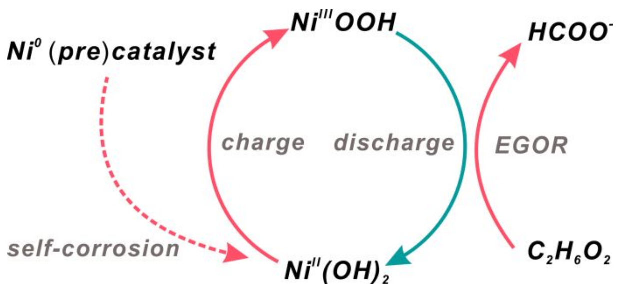

Figure S26. Schematic illustration of Ni species evolution during EGOR.

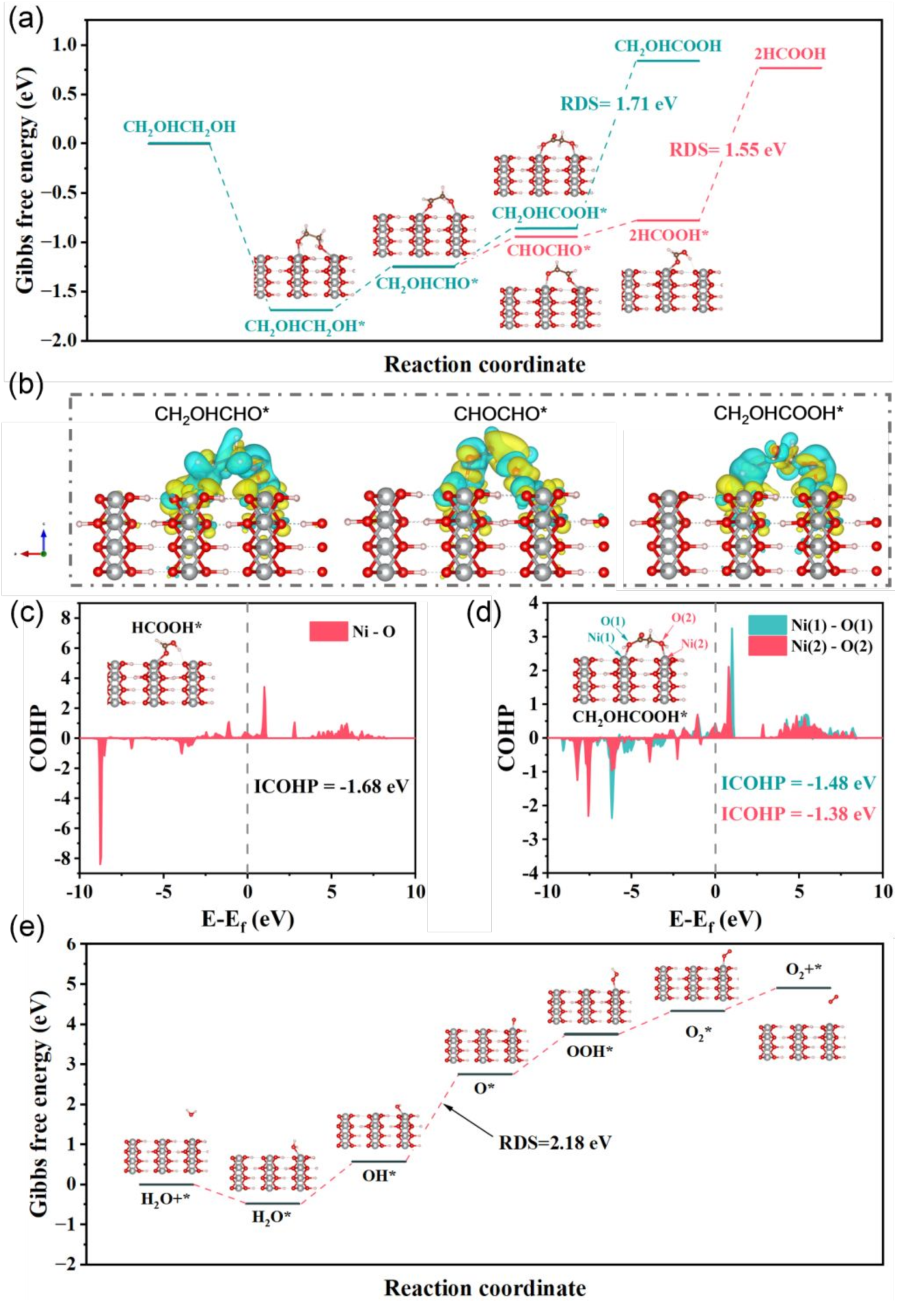

Figure S27. (a) Gibbs free energy diagram and reaction pathway of EGOR on NiOOH. (b) Schematic

illstration of charge density difference (CDD) for CH_2OHCHO*, CHOCHO*, and $CH_2OHCOOH$. Crystal orbital Hamilton population (COHP) of Ni 3d and O 2p for (c) HCOOH* and (d) $CH_2OHCOOH$* on NiOOH. The models and atomic numbering are shown in the inserted figures. (e) Gibbs free energy diagram and reaction pathway of OER on NiOOH.

Note to Figure S27: Figure S27a shows EGOR on NiOOH follows the reaction pathway of CH_2OHCHO* to CHOCHO* since the energy barrier is lower than of CH_2OHCHO* to $CH_2OHCOOH$*.[9,10] Bader charge and charge density difference in panel (b) indicate a charge-transhfer number of 0.136e for CH_2OHCHO* to CHOCHO*, which is higher than that of CH_2OHCHO* to $CH_2OHCOOH$* (0.026e). This result confirms that the EGOR pathway toward formate, as illustrated in panel (a), is more favorable. The rate- determing step (RDS) of EGOR on NiOOH (for both formate and glycolate products) is identified as the desorption step. Orbital interaction between Ni 3d and O 2p was characterized by ICOHP (integrated crystal orbital Hamilton population). Due to the stronger bridging adsorption of $CH_2OHCOOH$ on NiOOH, it shows a higher binding energy for $CH_2OHCOOH$* (-2.86 eV) than HCOOH* (-1.68 eV) (see panels c and d).[11] The lower energy barrier for desorption determines the high selectivity of formate. In panel (e), the Gibbs free energy for OER is calculated and shows a higher energy barrier (RDS=2.18 eV) compared with EGOR (RDS=1.55 eV for formate), evidencing the thermodynamic favorability of EGOR over OER.[5]

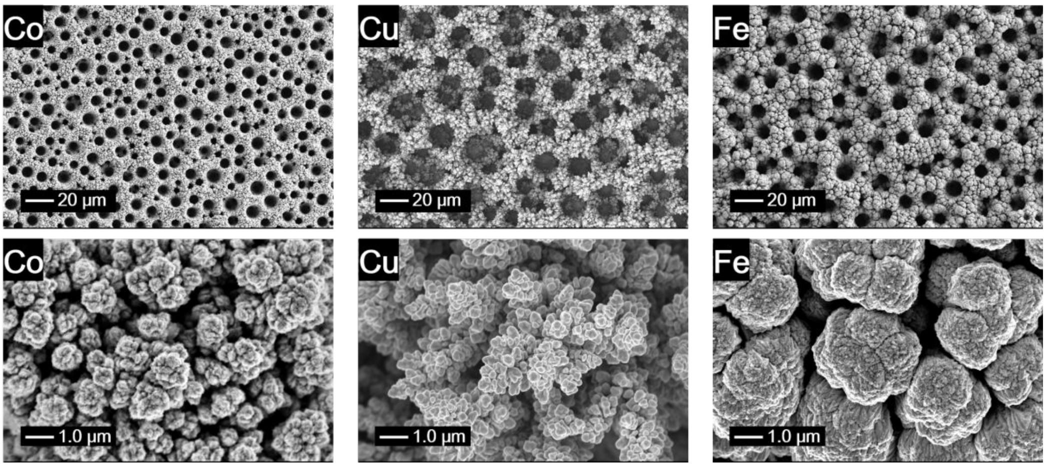

Figure S28. SEM images of the metal foams (Co, Cu, and Fe) deposited using the DHBT method.

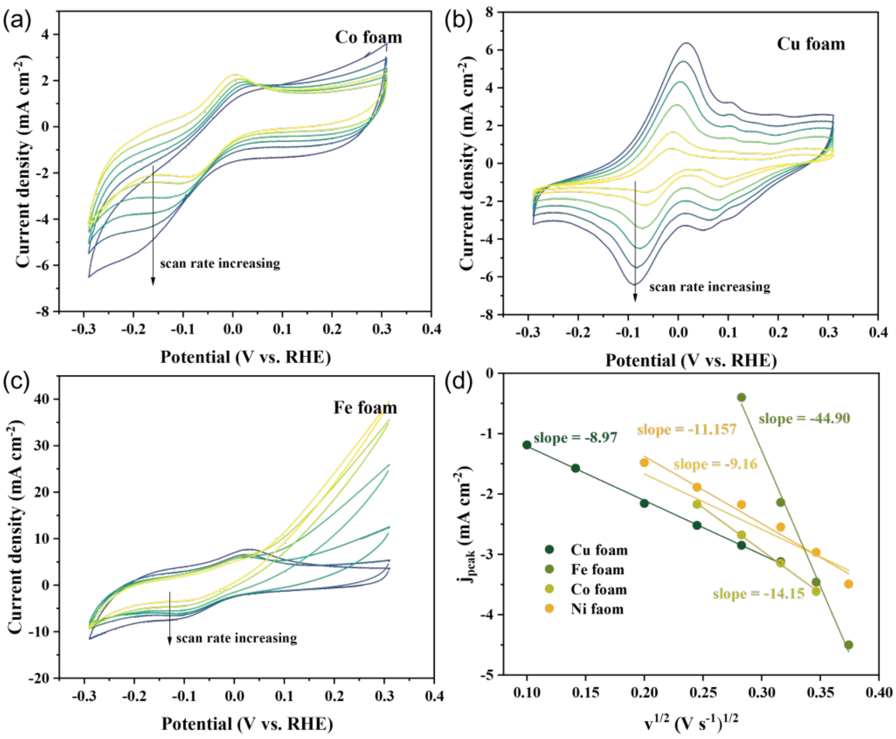

Figure S29. Cyclic voltammetry curves of (a) Co foam, (b) Cu foam, and (c) Fe foam in 10 mM $DMVCl_2$ + 0.5 M Na_2SO_4. (d) Corresponding fitting results based on R-S equation (Eq. 1).

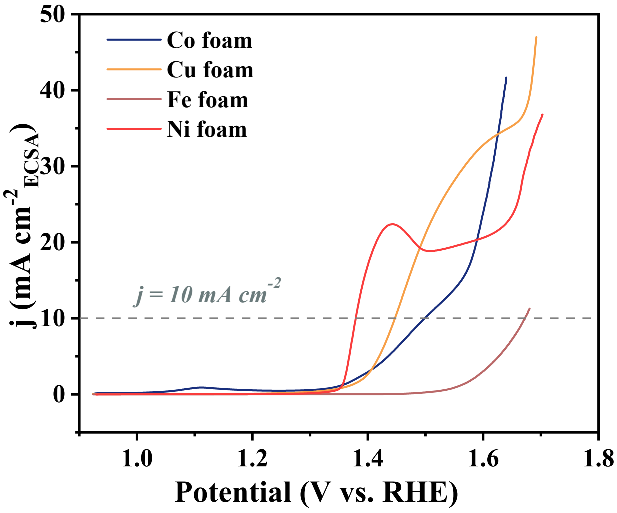

Figure S30. Cyclic voltammograms of different metal foams in 1M KOH + 0.1 M EG, with a sweep rate of 10 mV s^{-1}. The current densities were normalized by their ECSA while the potential was 85% iR-corrected.

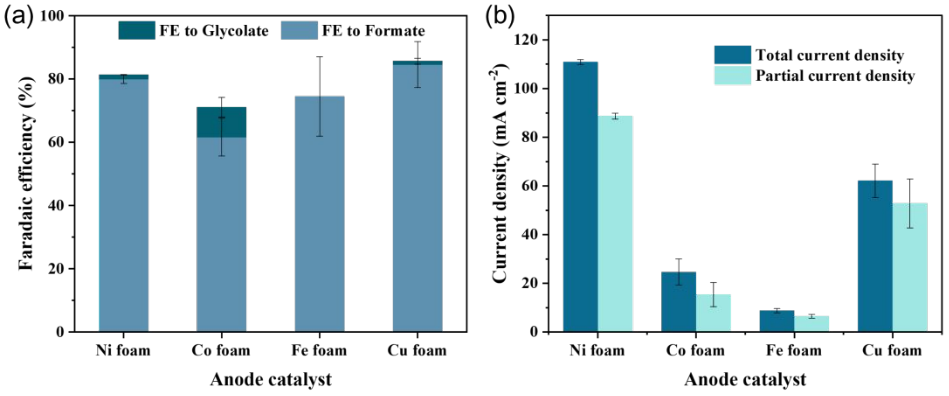

Figure S31. Electrolysis results of metal foams: (a) Faradaic efficiency to formate and glycolate and (b) averaged total/partial current density. The potentiostatic electrolysis was carried out at 1.5 V vs. RHE for 30 min with 100% online iR-compensation, and the current densities were normalized to their ECSA.

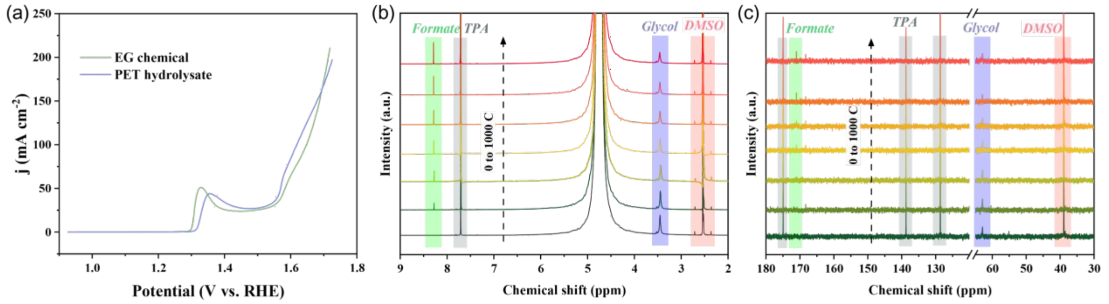

Figure S32. (a) LSV curves in EG monomer solution and PET hydrolysate, (b)^{1}H and (c) ^{13}C-NMR results of PET hydrolysate with different applied charges, in which characteristic peaks in gray, purple, green and pink represent TPA, EG, formate and internal standard (DMSO), respectively.

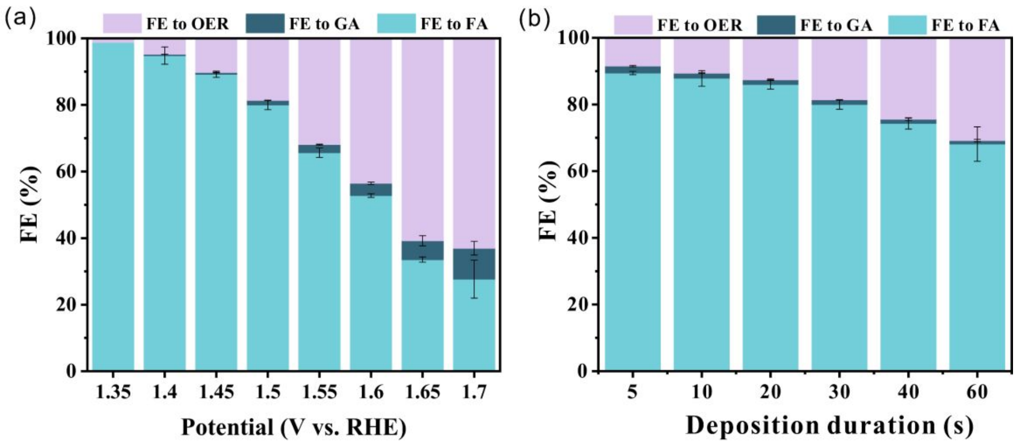

Figure S33. Faradaic efficiency acquired from potentiostatic electrolysis in H-type cell: (a) potential dependent with Ni foam@foil 30 s and (b) different deposition durations at 1.5 V vs. RHE. Ni foam@foil was applied as working electrode and 1.0 M KOH+100 mM EG as electrolyte. The electrolysis process was performed with constant charge of 360 C and 100% online iR-compensation.

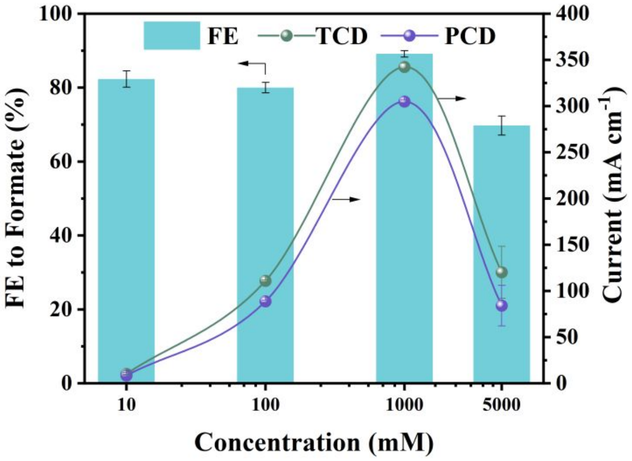

Figure S34. Faradaic efficiency and current results with different EG concentrations, at the potential of 1.5 V vs. RHE for 30 min. 1.0 M KOH acted as the supporting electrolyte in the H-type cell.

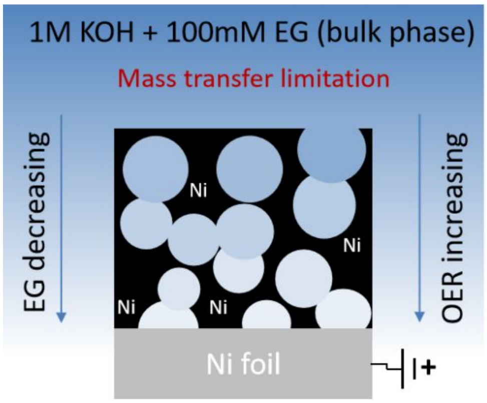

Figure S35. Illustration of the diffusion effect on the interface of Ni foam and electrolyte. EG depletion happens to the inner layer of Ni foam due to its fast oxidation kinetics, where oxygen evolution becomes dominant. The overall FE of EGOR is therefore constrained with thicker foam layers.

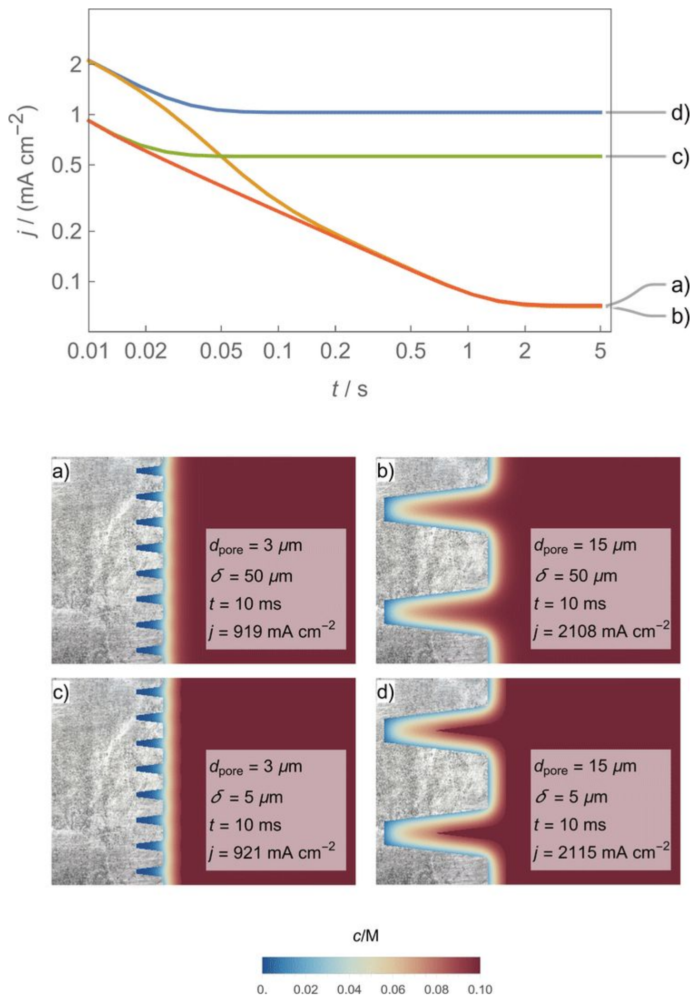

Figure S36. Calculated reactant (EG) concentration profiles are shown in the form of heat maps along foam electrode surfaces with different characteristic pore sizes d_{pore}, for different values of the diffusion layer thickness δ.

Note to Figure S36: In Figure S36, current transients (top part) and corresponding near-electrode reactant depletion scenarios (colored panels) are shown for the studied four d_{pore} / δ combinations. Of note, all the studied foams have the same geometric *and* full physical surface area (in a ratio of approximately 1:3), independent of the pore size. Nonetheless, the initial sections of the current transients clearly show that the large effective area can, in absence of convection, only be upheld temprarily (see panels and curves a and b), and increasing the pore size will only postpone in time, but eventually not avoid, the depletion of pores. On the other hand, in case convection is applied (suppressing the value of δ from 50 to 5 μm, see panels c and d), foam electrodes with larger openings —that allow the penetration of the diffusion layer into the pores— will be able to deliver higher current due to a better utilization of the electrochemically active surface area.

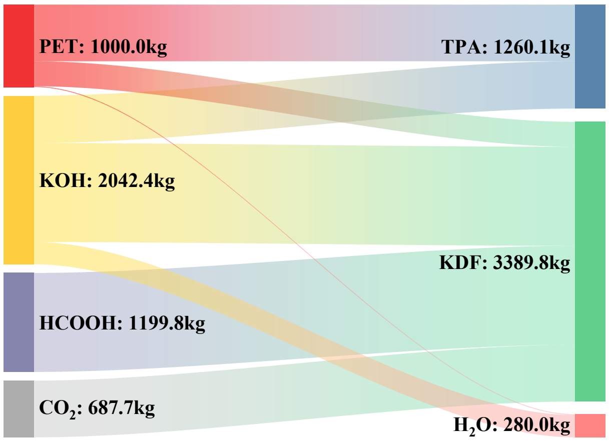

Figure S37. Material balance of the overall process. Waste PET served as the precursor of the electrolysis substrate (EG), KOH as the medium of hydrolysis and electrolysis, HCOOH as acidifying agent, and CO_2 as the cathodic feedstock, while TPA and KDF were obtained as the main products.

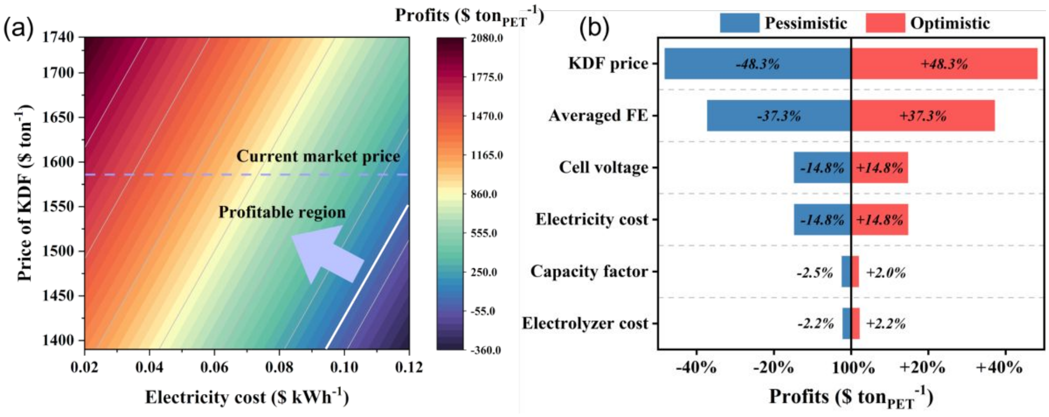

Figure S38. (a) Profitability assumption as a function of KDF price and electricity cost, (b) sensitivity ranking of the integrated electrolysis process. Note that the average FE in panel (b) was determined as a weighted average according to the electrons involved in the anode ($3e^-$) and cathode ($2e^-$).

Performance metrics and comparison to literature

Table S1. Summary and comparison of EGOR performance metrics of recently reported non-noble metal catalysts and this work.

Catalyst	Electrolyte	Formate yield (mmol cm^{-2} h^{-1} at given current density)	Stability	Ref.
Co-Ni_3N	0.1M EG +1M KOH	N/A at 50 mA cm^{-2}	12 hours at 50 mA cm^{-2}	[12](2023)
$CoNi_{0.25}P$	0.3M EG + 1M KOH	5.1 at 500 mA cm^{-2}	39 hours at 350 mA cm^{-2}	[13](2021)
B,Co-NiS	0.1M EG + 1M KOH	4.25 at 300 mA cm^{-2}	48 hours at 300 mA cm^{-2}	[14](2023)
$Ni(OH)_2$	PET hydrolysate + 10M KOH	3.3 at 500 mA cm-$^{-2}$	40 hours at 500 mA cm^{-2}	[15](2023)
FeCo–Ni_2P	PET hydrolysate + 1M KOH	N/A at 280 mA cm^{-2}	30 hours at 10 mA cm^{-2}	[16](2024)
Amorphous NiO	0.3M EG + 1M KOH	7.16 at 1000 mA cm^{-2}	10 hours at 1000 mA cm^{-2}	[3](2025)
Ni–Co_9S_8	1M EG + 1M KOH	N/A at 140 mA cm^{-2}	24 hours at 100 mA cm^{-2}	[17](2024)
NiCu	0.3M EG + 1M KOH	0.7 at 250 mA cm^{-2}	24 hours at 10 mA cm^{-2}	[18](2024)
$NiCo_2O_4$	0.5M EG + 1M KOH	2.68 at 230 mA cm^{-2}	50 hours at 200 mA cm^{-2}	[19](2025)
MoS_2@NiVFe-LDH	1M EG + 1M KOH	N/A at 200 mA cm^{-2}	60 hours at 20 mA cm^{-2}	[20](2025)
Mo-$Ni(OH)_2$	Alkaline seawater + 0.5 M EG	N/A at 300 mA cm^{-2}	300 hours at 100 mA cm^{-2}	[21](2025)
CoNiOOH–Ni_3S_2	0.5M EG + 1M KOH	3.14 at 280 mA cm^{-2}	10 hours at 120 mA cm^{-2}	[22](2025)
NiFe-LDH	0.5M EG + 1M KOH	N/A at 100 mA cm^{-2}	24 hours at 10 mA cm^{-2}	[23](2025)
**3D Ni foam**	**1M EG +1M KOH**	**10.5 at 1200 mA cm^{-2}**	**100 hours at 500 mA cm^{-2}**	**This work**

Table S2. Summary and comparison of EGOR + CO_2RR performance metrics of recently reported catalysts and the present work.

Anode	Cathode	Cell type	Formate yield (mmol cm^{-2} h^{-1} at given current density)	Stability	Ref.
CuCoO	$Bi_2O_2CO_3$	flow	N/A at 25 mA cm^{-2}	24 hours at 25 mA cm^{-2}	[24](2023)
$Ni(OH)_2$-VO	Bi/Bi_2O_3	flow	N/A at 100 mA cm^{-2}	4.2 hours at 100 mA cm^{-2}	[25](2023)
CuO@$Ni(OH)_2$	Pb-SnO	flow	0.062 at 20 mA cm^{-2}	24 hours at 20 mA cm^{-2}	[26](2024)
Mn/CoOOH	OV-rich BOC	flow	0.138 at 200 mA cm^{-2}	100 hours at 150 mA cm^{-2}	[27](2025)
NiOOH/NF	Bi_2O_3	MEA	N/A at 100 mA cm^{-2}	2 hours at 100 mA cm^{-2}	[28](2026)
NiOOH/$Ni_3Bi_2S_2$	Bi_2S_3	MEA	N/A at 400 mA cm^{-2}	50 hours at 400 mA cm^{-2}	[29](2024)
NiMn-LDH	InO_x	flow	5.2 at 200 mA cm^{-2}	100 hours at 100 mA cm^{-2}	[30](2025)
SnO_2	In_2O_3	flow	N/A at 180 mA cm^{-2}	30 hours at 120 mA cm^{-2}	[31](2025)
$NiCo_2O_4$	BiOI-C	MEA	N/A at 250 mA cm^{-2}	140 hours at 250 mA cm^{-2}	[32](2025)
**3D Ni foam**	**$Bi_2O_2CO_3$**	**flow**	**30.1 at 1200 mA cm^{-2}**	**100 hours at 500 mA cm^{-2}**	**This work**

Economic feasibility analysis

To assess the economic feasibility of the PET upcycling process, we performed a simplified techno-economic analysis (TEA) using a model adapted from those reported by Duan[13] and Resasco[33]. The plant was assumed to process 100 tons of waste PET per day. Plant lifetime was assumed for 10 years. Prices of feedstocks and products are provided in Table S3.

Table S3. Price of feedstocks and products. Listed references:

Products	Market Price ($/ton)	Ref.
Waste PET	374.00	a
KOH	750.00	b
EG	760.00	c
Formic acid	820.00	d
CO_2	220.00	e
TPA	1260.00	f
KDF	1590.00	g

a. https://resource-recycling.com/plastics/2025/05/21/bale-pricing-bucks-seasonal-trend-trade-alliances-shift/
b. https://businessanalytiq.com/procurementanalytics/index/potassium-hydroxide-price-index/
c. https://businessanalytiq.com/procurementanalytics/index/ethylene-glycol-price-index/
d. https://businessanalytiq.com/procurementanalytics/index/formic-acid-price-index/
e. https://www.imarcgroup.com/liquid-carbon-dioxide-pricing-report
f. https://businessanalytiq.com/procurementanalytics/index/terephthalic-acid-price-index/
g. https://jskolod.en.made-in-china.com/product/aFlJndtVYuch/China-Manufacturer-Supply-Feed-Grade-Potassium-Diformate-98-.html

Table S4. Assumptions for the sensitivity analysis.

	Optimistic	Basic	Pessimistic
Electricity cost ($/kWh)	0.054	0.06	0.066
KDF price ($/ton)	1749	1590	1431
Electrolyzer cost ($/m^2)	9000	10000	11000
Averaged FE	0.99	0.9	0.81
Cell voltage (V)	4.293	4.77	5.247
Capacity factor	0.99	0.9	0.81

The following assumptions were applied in the analysis:

1. Electrolyzer capital cost was assumed to scale with operating current density, with a baseline cost of 10,000 \$/m². Catalyst and membrane costs were set at 5% of the electrolyzer cost.
2. Hydrolyzer and separation unit costs were assumed to be 50% of the electrolyzer cost.
3. Plant utilization was represented by a capacity factor of 0.9, equivalent to 21.6 operating hours per day.
4. Material balance considered PET, KOH, formic acid, and water as inputs, with KDF and PTA as products. The purity of waste PET was assumed to be 90%.
5. Electrochemical parameters including Faradaic efficiency and cell voltage were collected from the real electrolysis under corresponding conditions. This assumption was based on the current density of 600 mA cm^{-2}, and the corresponding FEs of formate are 85% at anode and 94% at cathode, respectively. The cell voltage is 4.77 V.
6. Electricity cost was assumed to be 0.02-0.12/kWh regarding the renewable electricity from different sources. Energy demand was divided into three components: (i) PET hydrolysis, (ii) EGOR+CO_2RR paired electrolysis, and (iii) product separation.
7. Operation and maintenance (O&M) costs were set at 10% of total electricity costs.

The calculation steps are summarized below:

1. *Capital Costs*

Electrolyzer area is determined by current density: Area = I / j × 3.5, where the coefficient 3.5 was taken due to the asymmetric electrode design (anode:cathode area = 1:6).
Electrolyzer cost = Area × 10,000 \$/m²
Catalyst and membrane cost = 5% × Electrolyzer cost
Hydrolyzer and separation = 50% × Electrolyzer cost
Capital cost per day = (Electrolyzer cost + Catalyst and membrane cost + Hydrolyzer and separation) / (10×365)

2. *Maintenance Costs*

Maintenance cost per day = 10% × Capital cost

3. *Balance of Plant*

Balance of plant cost = 10% × Capital cost

4. *Installation Costs*

Installation cost = 10% × Capital cost

5. *Electricity Costs*

Charge required: Q = Plant capacity × F × n / M_{PET} / FE)
where the Plant capacity is 100 ton/day, F = 96485 C/mol refers to Faraday constant, n = 6 is the electrons needed for EGOR, M_{PET} = 192 g/mol is the molar weight of PET monomer, and FE refers to the Faradic efficiency of anode.

Current required: I = Q / (Operating time × Capacity factor)
Power consumption: P = (U × I)/1000 (kW)
Daily energy use: E = P × Operating time × Capacity factor
Electrolyzer electricity cost = E × Electricity price
Hydrolysis & separation electricity cost = 10% × Electrolyzer electricity cost
Total electricity cost = Electrolyzer electricity cost + Hydrolysis & separation electricity cost

6. *Input chemical*

The cost of feedstock was calculated according to the material balance (Figure S37) and prices (Table S3)
Input chemical cost = Σ (Unit price × Mass required)

7. *Operating Costs*

Operating cost per day = 10% × Total electricity cost

8. *Total Cost*

Total cost = Capital + Balance of plant + Installation + Maintenance + Input chemicals + Operating + Electricity

9. *Product Value and Profitability*

Product yields and corresponding prices were presented in Figure S37 and Table S3, respectively.
Product value = Σ (Unit price × Mass produced × yield ratio), where the yield ratio is taken as 80%
Net profit per day = Product value − Total cost
Payback period = Capital cost / Net profit / 365 (year)

Supplementary References

(1) Vesztergom, S. A Short Introduction to Digital Simulations in Electrochemistry: Simulating the Cottrell Experiment in NI LabVIEW. *J. Electrochem. Sci. Eng.* **2018**, 8 (2), 171–181.

(2) Jiang, X.; Zhao, K.; Feng, H.; Ke, L.; Wang, X.; Liu, Y.; Li, L.; Sun, P.; Chen, Z.; Sun, Y.; et al. Unraveling Side Reactions in Paired CO2 Electrolysis at Operando Conditions: A Case Study of Ethylene Glycol Oxidation. *J. Am. Chem. Soc.* **2025**, 147 (16), 13471–13482.

(3) Ding, W.; Ji, D. X.; Wang, K. K.; Li, Y. H.; Luo, Q. L.; Wang, R. W.; Li, L. L.; Qin, X. H.; Peng, S. J. Rapid Surface Reconstruction of Amorphous–Crystalline NiO for Industrial-Scale Electrocatalytic PET Upcycling. *Angew. Chem., Int. Ed.* **2025**, 64 (6), e202418640.

(4) Li, Y.; Ren, P.; Lu, X.; Zhang, J.; Yang, P.; Yang, X.; Wang, G.; Liu, A.; Wu, G.; An, M. Elucidating the Role of P on Mn- and N-Doped Graphene Catalysts in Promoting Oxygen Reduction: Density Functional Theory Studies. *SusMat* **2023**, 3 (3), 390–401.

(5) Wang, W.; He, X.; Tu, Z.; Xiong, D.; Dong, S.; Zhang, T.; Wu, D.; Wang, J.; Chen, Z. Spontaneous Electronic Redistribution Strategy for Efficient Electrocatalytic Upcycling PET (Polyethylene Terephthalate) and Nitrate Wastes. *ACS Catal.* **2025**, 15 (11), 9574–9583.

(6) Kresse, G.; Furthmüller, J. Efficient Iterative Schemes for ab initio Total-Energy Calculations Using a Plane-Wave Basis Set. *Phys. Rev. B* **1996**, 54 (16), 11169–11186.

(7) Fan, M.; Zhu, L.; Li, R.; Jiang, J.; Li, Y.; Wu, Y.; Ren, P.; Xu, H.; Wang, D.; Zhang, J.; An, M.; Yang, P. Engineering Metal–Support Interaction for Manipulate Microenvironment: Single-Atom Platinum Decorated on Nickel–Chromium Oxides toward High-Performance Alkaline Hydrogen Evolution. *Adv. Funct. Mater.* **2025**, 35, 2416678.

(8) Grimme, S. Semiempirical GGA-Type Density Functional Constructed with a Long-Range Dispersion Correction. *J. Comput. Chem.* **2006**, 27, 1787–1799.

(9) Wu, Y.; Ren, P.; Li, R.; Zheng, X.; Bai, C.; Liu, Y.; Meng, F.; Fan, M.; Peng, X.; Xiao, L.; Liu, A.; Xu, H.; Wang, D.; Zhang, J.; An, M.; Wen, S.; Li, Y.; Yang, P. Investigation of the Structure–Activity Relationship of Phosphorus-Doped Fe–Cu@NC Catalysts: Exploring the Influence of Different Coordination Layers on Oxygen Reduction Reaction Activity. *J. Power Sources* **2025**, 631, 236302.

(10) Perdew, J. P.; Burke, K.; Ernzerhof, M. Generalized Gradient Approximation Made Simple. *Phys. Rev. Lett.* **1996**, 77 (18), 3865–3868.

(11) Nørskov, J. K.; Rossmeisl, J.; Logadottir, A.; Lindqvist, L.; Kitchin, J. R.; Bligaard, T.; Jónsson, H. *J. Phys. Chem. B* **2004**, 108 (46), 17886–17892.

(12) Liu, X.; Fang, Z.; Xiong, D.; Gong, S.; Niu, Y.; Chen, W.; Chen, Z. Upcycling PET in Parallel with Energy-Saving H2 Production via Bifunctional Nickel–Cobalt Nitride Nanosheets. *Nano Res.* **2023**, 16 (4), 4625–4633.

(13) Zhou, H.; Ren, Y.; Li, Z.; Xu, M.; Wang, Y.; Ge, R.; Kong, X.; Zheng, L.; Duan, H. Electrocatalytic Upcycling of Polyethylene Terephthalate to Commodity Chemicals and H2 Fuel. *Nat. Commun.* **2021**, 12, 4679.

(14) Chen, Z. J.; Wei, W.; Shen, Y. S.; Ni, B. J. Defective Nickel Sulfide Hierarchical Structures for

Efficient Electrochemical Conversion of Plastic Waste to Value-Added Chemicals and Hydrogen Fuel. *Green Chem.* **2023**, 25 (15), 5979–5988.

(15) Liu, K. S.; Wang, Y. X.; Liu, F. L.; Liu, C. X.; Shi, R.; Chen, Y. Selective Electrocatalytic Reforming of PET-Derived Ethylene Glycol to Formate with a Faraday Efficiency of 93.2% at Industrial-Level Current Densities. *Chem. Eng. J.* **2023**, 473, 145292.

(16) Li, Y.; Lee, L. Q.; Zhao, H.; Zhao, Y. X.; Gao, P. Q.; Li, H. Alcohol-Alkali Hydrolysis for High-Throughput PET Waste Electroreforming-Assisted Green Hydrogen Generation. *J. Mater. Chem. A* **2024**, 12 (4), 2121–2128.

(17) Ma, Y.; Li, L. M.; Tang, J. L.; Hu, Z. K.; Zhang, Y; Ge, H.; Jian, N.; Zhao, J.; Cabot, A.; Li, J. S. Electrochemical PET Recycling to Formate through Ethylene Glycol Oxidation on Ni–Co–S Nanosheet Arrays. *J. Mater. Chem. A* **2024**, 12 (48), 33917–33925.

(18) Kang, H. X.; He, D.; Yan, X. X.; Dao, B.; Williams, N. B.; Elliott, G. I.; Streater, D.; Nyakuchena, J.; Huang, J.; Pan, X. Q.; et al. Cu Promoted the Dynamic Evolution of Ni-Based Catalysts for Polyethylene Terephthalate Plastic Upcycling. *ACS Catal.* **2024**, 14 (7), 5314–5325.

(19) Jiang, M.; Yang, Y.; Wang, Y.; Wang, Y.; Ratova, M.; Wu, D. Closed-Loop Electro-Upcycling of PET Waste into Formate and Hydrogen via Self-Supported NiCo2O4 Spinel Arrays. *Green Chem.* **2025**, 27 (33), 9978–9991.

(20) Yang, S.; Han, J.; Bao, W.; Ai, T.; Wei, X.; Jiang, P.; Deng, Z.; Zhang, J. NiVFe-LDH Nanosheets Reinforced MoS2 Heterogeneous Interface Design for Glycol-Assisted Water Electrolysis. *Fuel* **2025**, 388, 134482.

(21) Xiao, Z.; Guo, H.; Lv, F.; Lin, Z.; Sun, Z.; Sun, C.; Tan, Y.; Huang, Q.; Luo, M.; Guo, S. Geography-Guided Industrial-Level Upcycling of Polyethylene Terephthalate Plastics through Alkaline Seawater-Based Processes. *Sci. Adv.* **2025**, 11 (22), eadu8381.

(22) Sun, J.; Shi, B.; Dai, S.; Chu, L.; Wang, H.; Huang, M. Promoted *OH Adsorption Facilitates C–C Bond Cleavage for Efficient Electrochemical Upcycling of Polyethylene Terephthalate. *ACS Catal.* **2025**, 15 (1), 529–542.

(23) Jiang, Y.; Li, J.; Guo, X.; Chen, Y.; Sun, W.; Peng, C. Electrocatalytic Reforming of Polyethylene Terephthalate Waste Plastics into High-Value-Added Chemicals with Green Hydrogen Generation. *J. Colloid Interface Sci.* **2025**, 685, 29–37.

(24) Kilaparthi, S. K.; Addad, A.; Barras, A.; Szunerits, S.; Boukherroub, R. Simultaneous Upcycling of PET Plastic Waste and CO2 Reduction through Co-Electrolysis: A Novel Approach for Integrating CO2 Reduction and PET Hydrolysate Oxidation. *J. Mater. Chem. A* **2023**, 11 (47), 26075–26085.

(25) Ma, F.; Li, Z.; Hu, R.; Wang, Z.; Wang, J.; Li, J.; Nie, Y.; Zheng, Z.; Jiang, X. Electrocatalytic Waste-Treating-Waste Strategy for Concurrently Upgrading of Polyethylene Terephthalate Plastic and CO2 into Value-Added Formic Acid. *ACS Catal.* **2023**, 13 (21), 14163–14172.

(26) Bashir, I.; McGettrick, J. D.; Kühnel, M. F.; Sarfraz, B.; Arshad, S. N.; Rauf, A. Sustainable Formate Synthesis: Integrating Ethylene Glycol Oxidation with Carbon Dioxide Electrocatalysis Using Redox-Stabilized Earth-Abundant Electrodes. *ACS Sustainable Chem. Eng.* **2024**, 12 (12), 4795–4802.

(27) Liu, H.; Wang, Z.; He, Y.; Hu, X.; Liu, L. Simultaneous Electrochemical Upgrading of Polyethylene

Terephthalate Plastic and Carbon Dioxide into Valuable Chemicals. *Appl. Catal. B* **2025**, 361, 124667.

(28) Fang, Y.; Cai, C.; Yamashita, H.; Qian, X.; Zhao, Y. Efficient and Cost-Effective Electrocatalysts for Coproduction of Formate through Electrocatalytic Oxidation of PET-Derived Ethylene Glycol Coupled with CO2 Reduction. *Catal. Today* **2026**, 462, 115544.

(29) Hao, S. J.; Cong, M. Y.; Han, Z. Z.; Xu, H. W.; Liu, T. L.; Guo, M. X.; Ding, X.; Gao, Y. Electrocatalytic Formate Synthesis from Polyethylene Terephthalate and Carbon Dioxide through Sulfide-and-Reconstruct Engineering of Catalyst. *Chem. Eng. J.* **2024**, 498, 155106.

(30) Li, B.; Zhong, Z.; Li, H.; Yue, M.; Niu, Q.; Liu, L.; Xie, W.; Li, M.; Shao, M.; Wang, Q. Engineering Crystalline/Amorphous Interfaces for Enhanced CO2 Electroreduction. *Angew. Chem., Int. Ed.* **2025**, 64 (34), e202509502.

(31) Qiu, J.; Jing, Z.; Zhan, D.; Peng, J. Boosted Production of Formate via Paired Electro-Reforming of Poly(ethylene terephthalate) Plastic and CO2 Reduction. *Chem. Eng. J.* **2025**, 519, 165447.

(32) Sui, P.-F.; Zhu, M.-N.; Gao, M.-R.; Wang, Y.-C.; Feng, R.; Wang, X.; Liu, S.; Luo, J.-L. Energy-Conservative CO2 Electroreduction for Efficient Formate Co-Generation. *Appl. Catal. B* **2025**, 373, 125355.

(33) da Cunha, S. C.; Resasco, J. Insights from Techno-Economic Analysis Can Guide the Design of Low-Temperature CO2 Electrolyzers toward Industrial Scaleup. *ACS Energy Lett.* **2024**, 9 (11), 5550–5561.
